# The COPD Knowledge Base: enabling data analysis and computational simulation in translational COPD research

**DOI:** 10.1186/1479-5876-12-S2-S6

**Published:** 2014-11-28

**Authors:** Isaac Cano, Ákos Tényi, Christine Schueller, Martin Wolff, M Mercedes Huertas Migueláñez, David Gomez-Cabrero, Philipp Antczak, Josep Roca, Marta Cascante, Francesco Falciani, Dieter Maier

**Affiliations:** 1 Hospital Clinic - Institut d'Investigacions Biomediques August Pi i Sunyer (IDIBAPS).Universitat de Barcelona, 08036 Barcelona, Spain; 2 Biomax Informatics AG, 82152, Planegg, Germany; 3 Department of eHealth, Barcelona Digital, Roc Boronat 117, 08017 Barcelona, Catalunya, Spain; 4 Unit of computational Medicine, Center for Molecular Medicine, Department of Medicine, Karolinska Institute and Karolinska University Hospital, SE-171 76 Stockholm, Sweden; 5 Computational Systems Research, University of Liverpool, Liverpool L69 7ZB, UK; 6 Centro de Investigación Biomédica en Red de Enfermedades Respiratorias (CIBERES), Bunyola, Balearic Islands; 7 Departament de Bioquimica i Biologia Molecular i IBUB, Facultat de Biologia, Universitat de Barcelona, 08028 Barcelona, Spain

## Abstract

**Background:**

Previously we generated a chronic obstructive pulmonary disease (COPD) specific knowledge base (http://www.copdknowledgebase.eu) from clinical and experimental data, text-mining results and public databases. This knowledge base allowed the retrieval of specific molecular networks together with integrated clinical and experimental data.

**Results:**

The COPDKB has now been extended to integrate over 40 public data sources on functional interaction (e.g. signal transduction, transcriptional regulation, protein-protein interaction, gene-disease association). In addition we integrated COPD-specific expression and co-morbidity networks connecting over 6 000 genes/proteins with physiological parameters and disease states. Three mathematical models describing different aspects of systemic effects of COPD were connected to clinical and experimental data. We have completely redesigned the technical architecture of the user interface and now provide html and web browser-based access and form-based searches. A network search enables the use of interconnecting information and the generation of disease-specific sub-networks from general knowledge. Integration with the Synergy-COPD Simulation Environment enables multi-scale integrated simulation of individual computational models while integration with a Clinical Decision Support System allows delivery into clinical practice.

**Conclusions:**

The COPD Knowledge Base is the only publicly available knowledge resource dedicated to COPD and combining genetic information with molecular, physiological and clinical data as well as mathematical modelling. Its integrated analysis functions provide overviews about clinical trends and connections while its semantically mapped content enables complex analysis approaches. We plan to further extend the COPDKB by offering it as a repository to publish and semantically integrate data from relevant clinical trials. The COPDKB is freely available after registration at http://www.copdknowledgebase.eu.

## Background

We previously reported the public availability of a chronic obstructive pulmonary disease (COPD) specific knowledge base [1]. This COPDKB semantically integrated existing COPD related knowledge such as genotype - phenotype relations or signal transduction pathways into structured networks that were connected with clinical and experimental data. To this end an object-oriented knowledge model was generated which contained concepts such as "gene", "disease" or "organ" and their associations such as "causes", "damages". We established a general human molecular knowledge network of over 3.6 million connections (e.g. gene-disease associations, protein-protein interactions) with disease-specific signal transduction (54 pathways) and metabolite (122) information manually curated from the literature. Initial search, retrieval and R-plugin -based data-mining methods integrated into the COPDKB enabled the retrieval of disease- or case-specific sub-networks e.g. lung specific by expert users for data analysis and model generation. To this end a thick Java client provided a wizard based user interface to create natural language like queries such as

"Object to find is a Patient which simultaneously is annotated by Patient diagnostic data which has GOLD attribute greater than 2 and is annotated by Patient Anthropometrics which has BMI-BT attribute less than 18 and never is diagnosed with a NCI Thesaurus entry which is inferred by ontology entry which has name like '*cancer*'", which would retrieve all patients diagnosed with COPD severity grade above 2 but no cancer which have low body mass index. In addition graph based navigation allowed single step network expansions to e.g. navigate from a group of patients to the diseases they are diagnosed with and from there to the genes associated with these diseases. However, validation with user groups showed that to enable application by clinical researchers, a significant simplification of the user interface was required.

To extend the applicability of the COPDKB as a central part of a biomedical research platform (see [2]) we identified several aims:

1. Update existing and integrate further COPD-specific knowledge and semantically map it to clinical, physiological and molecular data of COPD patients to generate a full repository of COPD-associated features.

2. Extend the capability of knowledge representation to include non-SBML-based mathematical models and integrate COPD-specific computational models. Semantically connect models of different types (e.g. ODE, probabilistic) with each other as well as existing relevant data.

3. Generate an intuitive browser-based user interface for clinical and biomedical researchers.

4. Connect the aggregated COPD-specific knowledge to a clinical decision support system (CDSS), which provides translation into clinical practice.

## Methods

**Figure 1:**
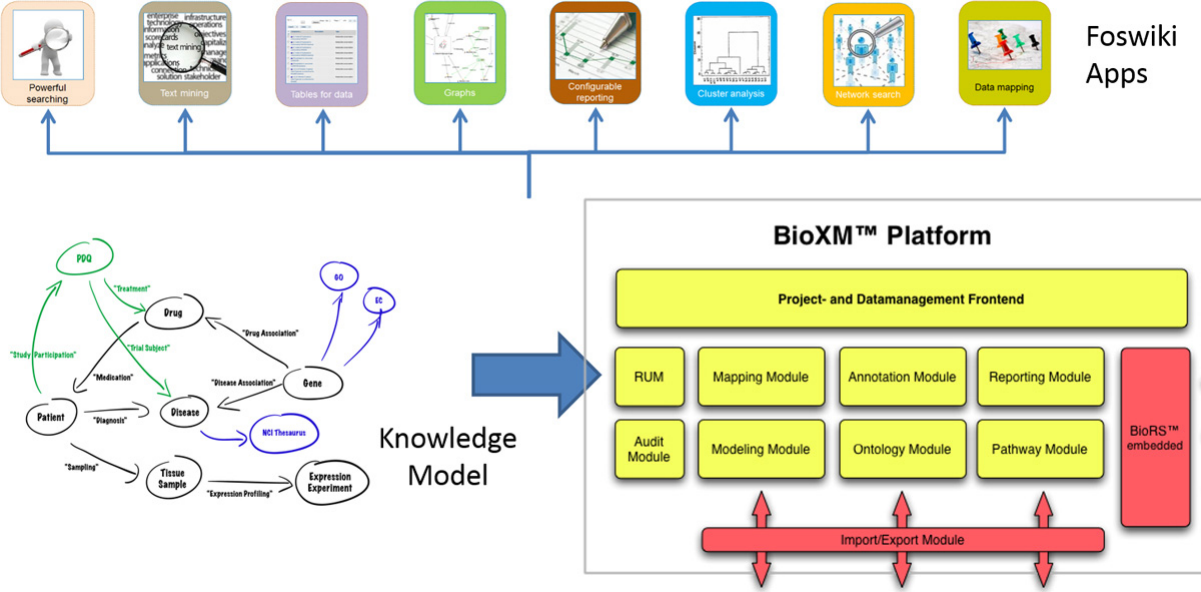
**BioXM module structure**. The modular BioXM architecture decouples tasks such as authorisation and access control from data management, search and reporting. The dynamic semantic knowledge model defines which objects and associations can be represented and analysed. Selecting from multiple "App" functions a highly specific browser-based user interface can be generated within the Foswiki open source framework.

The requirement specification used elicitation methods such as user observations, focus groups, interviews and workshops to establish the use cases and workflows, which were refined based on a prototyping approach applying agile development methods. The architecture design approach followed the ISO/IEC 42010:2007 standard. Implementation of the user interface framework is based on the open source Foswiki framework (http://www.foswiki.org) for which a plugin was developed to connect the BioXM™ Knowledge Management Environment. While details of the technical architecture of BioXM have been reported elsewhere [3] we briefly summarise it here to aid understanding. As depicted in Figure 1 BioXM is implemented as a platform-independent Java client-server application with modular architecture and a relational database management system backend. The Foswiki plugin calls a dedicated servlet deployed in an Apache Tomcat servlet container. The servlet connects to different BioXM application server programming interfaces (APIs) to execute searches and retrieve pre-defined reports through the wiki plugin into an html user interface which is accessible by any modern web-browser. A BioXM SOAP web service is used to interoperate with external applications such as the Clinical Decision Support System (CDSS) and Simulation Environment (SE). Data analyses methods are based on R scripts, which the BioXM R-plugin calls and presents the results directly in the user interface. Based on this plugin, content displayed in the browser can be dynamically generated from the knowledge base repository. The resulting user interface was iteratively validated and refined by focus groups of biomedical researchers within the Synergy-COPD projects as well as biohealth research students during two subsequent summer schools of the Erasmus Mundus BioHealth Computing program (see [4] ).

Data integration and semantic mapping was performed as described previously [1]. Briefly summarized, semantic mapping templates are generated manually between the conceptual data model of individual resources and the disease-specific knowledge model. Integration of data updates are subsequently performed automatically.

Manual curation of disease-specific knowledge was generated by extracting from expert-specified publications and the results were enriched by expert panel discussions.

## Results

### Overview of the knowledge base

The COPDKB now provides interoperability and integration between multiple data sources and tools commonly used in biomedical research. It also extends to include new tools such as a multi-scale Simulation Environment [5] that enables the execution of disease-specific simulations based on the integration of multiple sub-models. The COPDKB is based on the concept of "knowledge as network" and bridges multiple sources and scales of knowledge by abstracting commonly used concepts to communicate disease-specific knowledge into objects and their relations. Structuring explicit and implicit knowledge into these formal concepts enables the use of existing, well-defined vocabularies (e.g. GO[6], ICD10 [7]) and standards (e.g. SBML[8], HL7[9]) to represent molecular, biochemical and clinical processes.

One of the challenges for the use of computation models in biomedical research is the integration of models at different scales as well as the mapping to corresponding clinical, physiological or molecular data. We defined standard operating procedures for model documentation and developed a realisation for the concept of composite use of orthogonal ontologies [10] to create semantic descriptions for models, model parameters and clinical parameters. The concept of combinatorial ontology use. Traditionally, ontologies are created with the intention to establish well defined, highly detailed concepts that capture the full semantic meaning of complex facts such as "positive regulation by symbiont of defense-related host calcium-dependent protein kinase pathway (GO:0052102)". In this way such a fact can be expressed by assigning a single ontological term. However, due to the complexity and expressivity of descriptions for biomedical functions and processes especially in physiology often no single ontological concept will fully describe the semantic meaning. Therefore a combinatorial description which combines multiple concepts has to be created semi-automatically e.g. by matching a free text description such as "partial arterial blood pressure" to terms in existing ontologies and then selecting the appropriate concepts from several, ideally orthogonal i.e. non-overlapping, ontologies, to generate an overall complete representation such as "MESH:D010313 Partial Pressure; PubChem:977 Oxygen; FMA:83066 Portion of arterial blood". Our realisation of this concept included standards for the definition of spatio-temporal compartments to allow ontology-based model-model and model-data connection. As a rule concepts should be selected from within a single ontology but if this does not create a full semantic description concepts from different ontologies can be combined. New inference methods are required to make use of such combinatorial descriptions and we decided to implement a network similarity search approach, which treats individual descriptors as objects in a network and uses within-ontology as well as between-ontology relations to infer object equivalence. Specifically, our algorithm treats the collection of descriptors as a specific network (*semantic_descriptorA*) and searches all other existing descriptor collections (*semantic_descriptorX *to *semantic_descriptorY*) for "similar" networks. A *semantic_descriptor *is more similar to the input the more a) shared nodes (ontology entries); b) identical edges (connections between two ontology entries); c) similar nodes (ontology entries connected to the identical node by intra- and inter-ontology relationship, i.e., ontological inference) and d) similar edges (alternative edge classes connecting identical or similar nodes) are found. A similarity score is calculated by summing individual node/edge scores multiplied by the coverage, i.e., total score = Sum(individual scores) * coverage. Individual scores are defined as "1" for identical matches and scores for similar nodes/edges are derived by dividing 1 by the number of required steps to reach the "similar" object, i.e., a distance-based measure with diminishing contribution. The coverage is calculated as a fraction of objects in the input *semantic_descriptor *actually recovered in the search targets.

**Figure 2:**
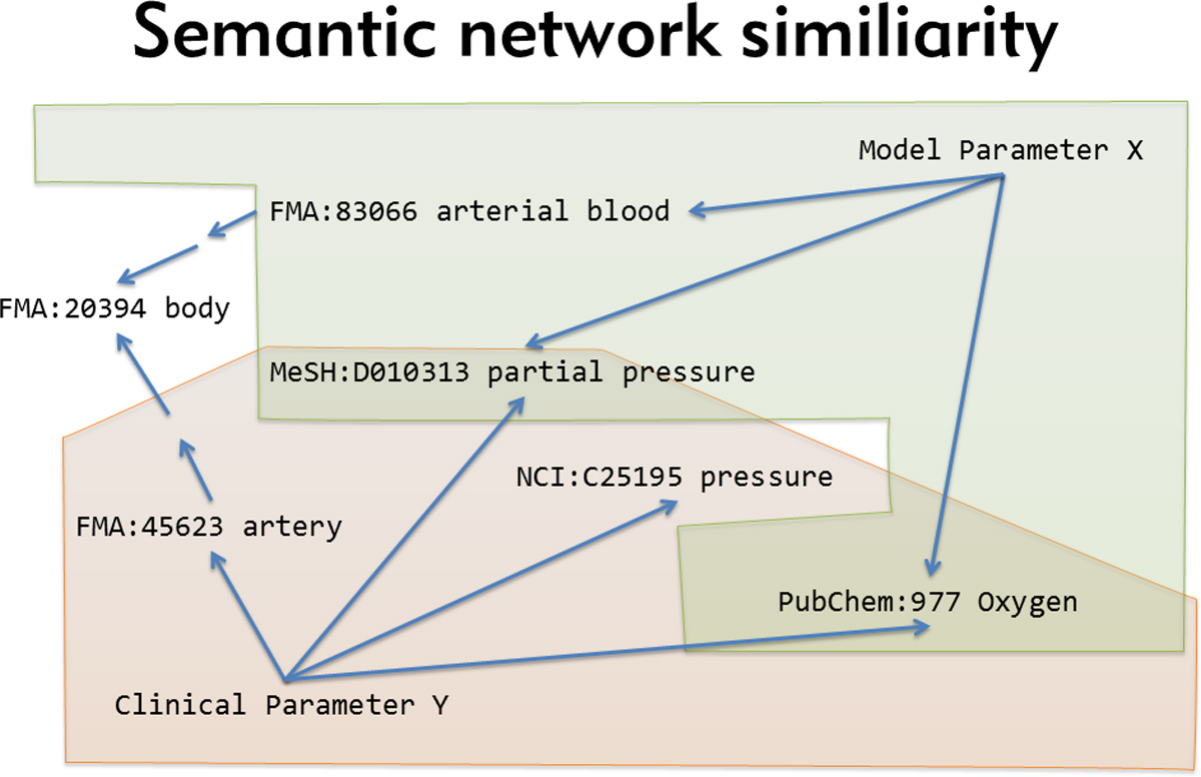
**Semantic network similarity**. "Equivalent" meaning of different parameters is determined by the overlap of their corresponding semantic descriptors, taking into account transitive relations within and between ontologies.

It is therefore possible to deduce, for example, a 98% similarity between the above described model parameter and a clinical parameter described as "NCI: C25378 Partial; NCI: C25195 Pressure; PubChem:977 Oxygen; FMA:45623 Systemic arterial system" (see Figure 2). The proposed mappings are verified manually and, if found true, are fixed, enabling the parameterisation of computational models based on personalised data, the integrative simulation of multi-scale models and the subsequent validation of model predictions against individual clinical data.

**Figure 3:**
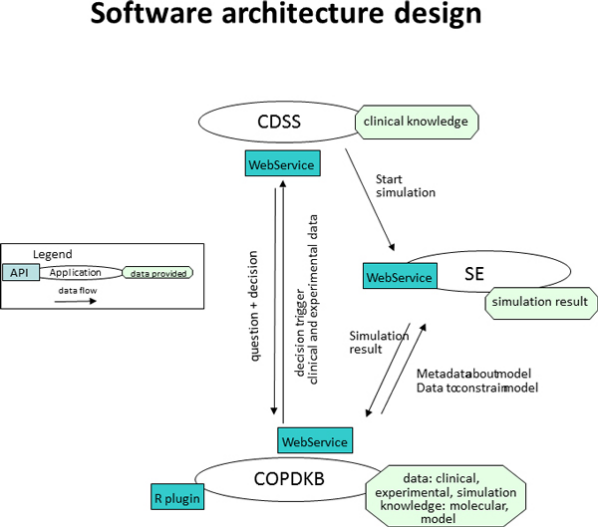
**Software architecture**. Integrative UML-like diagram representing the organisational, infrastructural and informational view of the software architecture. All modules communicate based on WebServices. The COPDKB provides information of type model, model paramters, parameter values (summarised as "simulation knowledge") and experimental data to the SE. The SE feeds back simulation results. With the CDSS the COPDKB exchanges requests for information on knowledge about co-morbidities and drug-interactions and provides corresponding search results. COPDKB = COPD Knowledge Base, SE = Simulation Environment, CDSS = Clinical Decision Support System, API = Application Programming Interface.

In addition to the semantic description of different types of knowledge and data, we therefore generated a technical integration between the three compounds required for integrative simulation and clinical decision support, the COPDKB, the Simulation Environment (SE, [5]) and Clinical Decision Support System (CDSS, [11]). We generated an architecture meta-model consisting of the following concepts: software components, graphical user interface (GUI), application programming interface (API), data types and information flow. Figure 3 shows an integrative UML-type diagram of the organisational, infrastructural and informational architecture views depicting the involved software components, their APIs and the information flows between them.

The user interaction was designed within the individual software applications to optimise the use-case and user-group-dependent issues. Therefore the COPDKB provides the primary access point to integrate, curate, search and retrieve COPD-related knowledge; the SE interface is designed to enable the explorative execution and personalisation of integrated, COPD-related computational models; and the CDSS ideally becomes unobtrusively integrated into the user interfaces of existing clinical information and management systems to extend the functionality of accepted clinical practice user interfaces to provide disease-specific, individualised support.

### Resources added to the COPDKB

We extended the disease-specific structured knowledge network significantly with several new resources (see Table 1 for an overview). Criteria for inclusion of new resources were the major aims of the Synergy-COPD project (see [2]): a) to analyse systemic, especially skeletal muscle related mechanisms involved in COPD b) to analyse co-morbidity related COPD heterogeneity and corresponding molecular mechanisms and finally c) to aid the generation of clinical decision support for diagnosis and therapy. To this end we applied literature mining, integration of existing structured databases, clinical studies and ontologies as follows:

**Table 1:** Additional resources integrated into the COPDKB.

Resource	Information type	Number of nodes	Number of associations
BioBridge omics network 2012	BioBridge 8w training extended by protein and metabolite measurements	4878	11448

Co-morbidity network	Co-morbidity network from OMIM gene-disease associations as well as Medicare and Swedish health system patient data	8 435	3 973 126

COPD literature mining	GWAS and epigenetic gene- disease associations	18	20

Disease grouping	Expert-based manual grouping of diseases	493 diseases	27 groups

Human angiogenesis young, old	Public, angiogenesis-related expression data	3691	3782

ICD 9	International classification of disease, ninth revision	13 222	

ICD 10	International classification of disease, tenth revision	12 416	

ITFP	Transcription factors and targets	2 143 TF, 6 710 targets	74 907

MeSH	Medical subject headings	24 767	

miRbase	micro RNA	2024 human miRNA	

miRTarBase	microRNA targets	12 194 human targets	38 311

Mouse inactivity-induced muscle wasting	Public mouse, inactivity-induced muscle wasting data gene expression GSE25908,	15 287	154 597

PAC-COPD	Clinical study	342 patients, 260 clinical attributes	

Pathway network	Jaccard index based clustering of pathways from KEGG, Reactome and the BioBridge COPD text mining	1138 pathways	1815 associations

SNOMED-CT	Systematized Nomenclature of Medicine -- Clinical Terms	296 519	

Data sources added since the first release of the COPDKB

1) Additional COPD-associated knowledge was curated from the literature. Within the Synergy-COPD project a main focus was on the understanding for epigenetic regulation of muscle phenotypes and development [12] as well as disease co-morbidities derived from OMIM gene-disease associations [13] and patient records [14].

2) The general knowledge on human regulatory processes was extended by integration of miRNA regulation information from miRBase [15] and transcriptional regulation information from ITFP [16].

3) Regarding clinical COPD data, we integrated a second major study on COPD, PAC-COPD [17], which focuses on the phenotypic heterogeneity and the extent to which this heterogeneity is related to clinical development of COPD.

4) We extended the range of ontologies integrated into the COPDKB to improve the coverage of medical terms, diagnosis and processes. To this end we integrated the Medical Subject Headings (MeSH, [18]), the International Classification of Diseases with Clinical Modifications, Ninth and Tenth Revision (ICD9-CM, ICD-10-CM) [19] and SNOMED [20]. We used the UMLS Metathesaurus [21] to derive mappings between the different medical vocabularies and managed to relate over 150 000 concepts. These mappings allowed us to integrate a number of different gene-disease association resources which had used different disease vocabularies e.g. OMIM or ICD9.

5) Finally the COPDKB was extended significantly by results derived from the Synergy-COPD project.

We updated the expression-based association network derived from the BioBridge clinical study [22] with a new version now integrating gene expression data with physiological attributes such as VO2max, protein modifications and metabolite measurements providing a COPD-training-specific network. A further three gene-expression-based association networks specific to angiogenesis in young and elderly adults as well as a mouse model on inactivity effects on muscle were made available (personal communication FF).

In addition to the general co-morbidity network mentioned above, two COPD-specific co-morbidity networks were generated within the project based on US Medicare and Swedish health system patient records totalling 13 million patients over 3 years [14] and 5 million patients over 9 years (personal communication DGC), respectively. To normalise health-system-specific differences in disease coding, a disease grouping was developed by clinical experts and integrated into the COPDKB. Similar to the disease grouping, we needed to bridge and unify signal transduction pathway, transcriptional regulation and metabolism information integrated from databases such as KEGG [23] and Reactome [24] as well as from COPD-specific literature mining efforts reported earlier [1]. To this end we generated a Jaccards index-based clustering based on the overlap of pathway participants. At a conservative cut off of 0% FDR, this resulted in 13 groups summarising 421 individual pathways (out of 1367 total). Finally, we integrated computational models describing different cells, tissues, organs and physiological processes involved in COPD and its systemic effects. Three of these form the core of a COPD disease model, specifically a lung air and blood flow model [25] an oxygen transport model [26-28] and a muscle cell bioenergetics and ROS production model [29,30].

Overall the COPDKB now provides access to almost 850 000 nodes, from genes, proteins and metabolites to cells, tissues and organs. 9.5 million associations between these nodes can be mined to derive COPD-specific hypotheses and data.

### Tools for mining

The simplest way to work with the updated COPDKB is by accessing its new, HyperText Markup Language (HTML) and browser-based user interface (see Additional file 1 for a detailed description and screenshots). It provides individual sections for disease-specific public knowledge, analysis results, mathematical and network models, as well as clinical data.

The browsing functions in each of these sections allow users to navigate through specific sub-sections e.g. the list of all COPD-associated genes or pathways.

All integrated information can be exported and can be filtered by keyword or numerical value using the column selector (e.g. "gene function: DNA binding" or "expression value: >1 AND < 100"). Cross-navigation between semantically mapped or associated data types is possible by following the "Change type" buttons, for example, to jump from a list of genes to the diseases associated with them. The "Data matrix" button allows users to show actual data associated to any displayed entity (if the user has access to those data, see above). On such a data matrix the "Statistics" button allows access to a simple box-plot overview statistic (mean, STDEV, min, max, quartiles) as well as t-test-based comparison of two groups (e.g. FEV1 for high/low BMI COPD patients).

**Figure 4:**
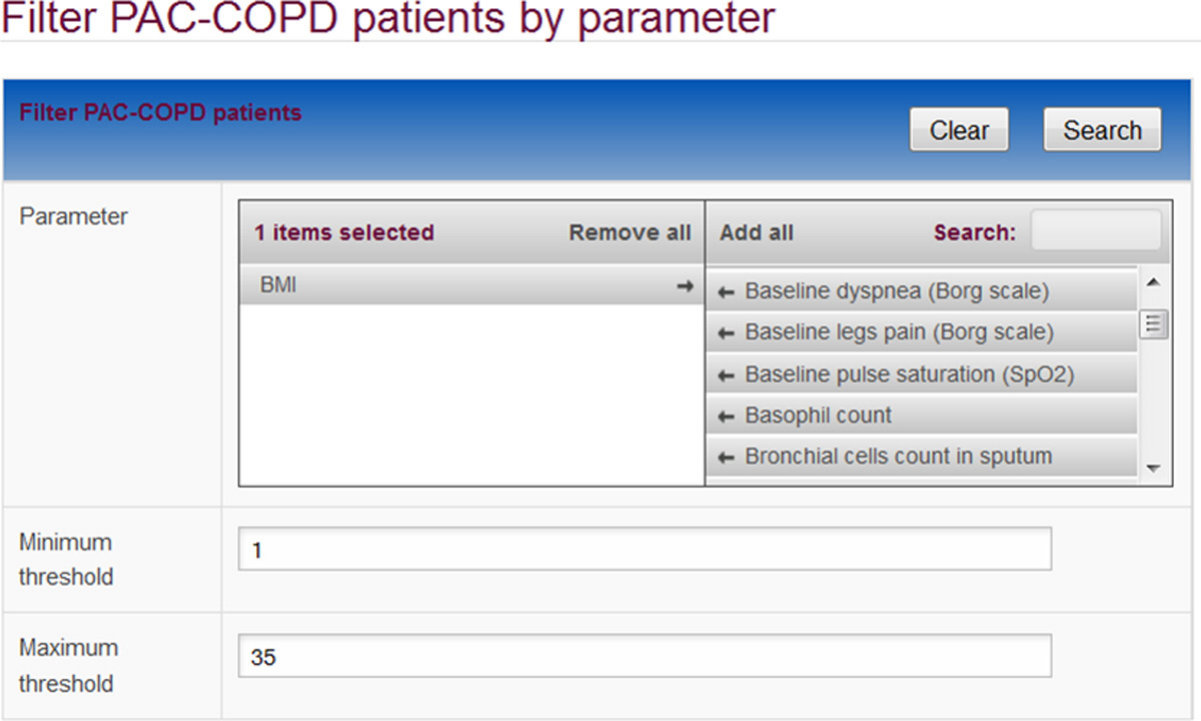
**Search form**. Search forms provide pre-defined selections of parameters for which thresholds can be set to retrieve data. Here the body mass index was selected to retrieve patients with corresponding values.

In addition to the simple browse and filter access, we implemented a set of form-based searches, which allow more direct access to information and are continuously expanded based on user feedback. Currently the search form for patient selection is based on all clinical parameters such as BMI or dyspnoea Borg scale, allowing minimum and maximum threshold values to be set (Figure 4). Other search forms allow molecular data (e.g. metabolite or protein measurements) to be retrieved.

Two interactive data-mining methods, the network search and network ranking, are currently available only from the Java-based expert user interface. These expert-generated results are then made available in the standard user interface in the same way as other data. The network search is a variant of the breadth-first-search [31], a graph search algorithm that begins at the root node and explores all the neighbouring nodes, which is iteratively repeated for each neighbour until a target node is reached. Within the COPDKB the path between nodes (the "associations") receives user-defined penalty scores and only the alternative with the lowest score or all alternatives below a certain threshold are retained. Different association types can carry different penalty scores. For example, a "high quality" protein-protein interaction (PPI) derived from co-immunoprecipitation might carry a penalty score of 1 while a "low quality" PPI detected by two-hybrid experiments might have a score of 3. Based on the overall penalty score, shorter and more "high quality" paths are preferred. The network ranking method developed within Synergy-COPD takes the result of a network search and assigns additional quality values to each of the nodes; these quality values can represent complex information such as "number of associated diseases" or "variability in muscle expression", the later derived from the analysis of over 4000 muscle-specific expression data sets available in GEO [32].

Finally the "network similarity" search is available directly from the standard COPDKB user interface. It compares different networks, e.g. signal transduction pathways or semantic descriptors of model parameters, based on the occurrence of identical or "similar" nodes and associations. In this context similarity is defined as proximity within an ontology. Based on this search method, a list of clinical and model parameters, for example, can be ranked according to their semantic similarity.

### Major uses in Synergy-COPD

So far the major use for the COPDKB has been as the central collaboration and biomedical research platform within the Synergy-COPD project. As Systems Medicine is an inherently interdisciplinary process, it is extremely important to enable the generation of a common language between experts from different disciplines. The COPDKB has been used to map between knowledge and data from clinical researchers, computer scientists and mathematicians using the semantic description concept and the network similarity search. Clinical parameters from two different clinical studies have been unified and integrated with three computational models and eight association networks. While the COPDKB currently provides only simple analysis features it has been extensively used to support complex analysis workflows. Integrative network association analysis on combined clinical and molecular (expression, metabolic, protein) data was enabled by the corresponding data mapping and integration in the COPDKB [22]. The connection between data analysis results and the existing computational models was derived from network searches which took the highly ranked genes/proteins/metabolites/clinical parameters from the data analysis and searched for connecting paths to the described model parameters based on, in case of the bioenergetics model [29,30], mappings to reference genes/proteins/metabolites. Another important type of information provided by the COPDKB were the multiple mapped and integrated gene-disease and gene-gene associations. These were mainly used to provide molecular connections and mechanisms between different diseases derived, for example, from co-morbidity analysis. As described separately [33], the COPDKB also forms the knowledge backbone for the Simulation Environment. It provides the mappings between different models that are required by the SE for integrative multi-scale model execution and it subsequently holds the simulation results and maps them back to corresponding clinical data for validation. The final major use case for the COPDKB is to act as a fact repository for the Clinical Decision Support System (CDSS, [11]). The COPDKB provides co-morbidity and drug-drug interaction information to the CDSS, which subsequently generates alerts on possible disease co-occurrences or adverse drug interactions.

A final important use case turned out to be using the COPDKB as an educational tool to introduce non-clinicians to the issues and challenges of COPD-related Systems Medicine. Within the Erasmus Mundus BioHealth Computing program [4] the COPDKB was the initial access point for all students to learn about the different aspects of Systems Medicine, from clinical question to available knowledge and data, to analysis methods and predictive mathematical models. The feedback provided by these focus groups in turn greatly helped to improve the COPDKB user interface and shape further requirements.

## Discussion

Within biomedical research we increasingly rely on computational support to keep track of our understanding of complex systems such as ecosystems or the human body and its malfunctions. So far, within Systems Medicine only a few examples of computational disease knowledge resources are available (e.g. [34]) and the COPDKB is to our knowledge the only such resource regarding COPD. Moreover, by integrating the COPDKB tightly with a Simulation Environment, it extends from a dynamic, but still lexicon-type reference resource, into a truly predictive and individual tool. Due to the integration with a Clinical Decision Support System it is able to deliver these individualised predictions directly into clinical practice.

However, several caveats remain. Although many of the available relevant structured public knowledge resources have been integrated, the majority of disease knowledge still remains hidden in the literature. Text-mining methods and manual curation provide some inroads into this wealth, but are far from sufficient to generate a truly complete picture of our current knowledge. Another limitation is the quality and context specificity of the integrated knowledge. Many resources do not extract these measures and therefore they remain hidden in the original literature. Detailed knowledge structuring efforts, such as developing a mathematical model of a certain process, still require major manual literature-review efforts; the COPDKB only provides a rough framework and an indication where to start and which pathways to follow.

Regarding a full biomedical research platform, the COPDKB currently still lacks accessibility of the integrated data-analysis and data-mining methods for non-expert users. The majority of available knowledge is on the molecular level and only a small part of it is specific to COPD.

## Conclusions

The COPDKB provides a step in our development of a biomedical research platform for System Medicine as well as the most comprehensive COPD-specific knowledge base. The COPDKB proved a valuable tool for the analysis and computational modelling of COPD although several gaps and weaknesses still remain, some inherent to the platform itself, some generic from the way we still communicate knowledge. Future developments will focus on three aspects: improving the quality of the disease-specific knowledge, extending the integrated COPD-related clinical data sources and bringing the validated data analysis workflows into the reach of non-bioinformaticians.

## Competing interests

DM, CS and MW are employed at Biomax Informatics AG and will therefore be affected by any effect of this publication on the commercial version of the BioXM™ Knowledge Management Environment on which the COPDKB is based. IC, AT, MH, DGC, PA, JR, MC and FF are employed at academic institutes and will therefore be affected by any effect of this publication on their publication records.

## Authors' contributions

IC conceived, implemented and validated the initial UI and co-drafted the manuscript. AT implemented the prioritisation strategy. CS specified the HL7 vMR support and implemented the medical ontology mapping. MW designed and implemented the technical UI framework. MH co-developed the COPDKB-SE integration architecture and implemented the integration interface. FF and PA and DGC co-developed the data analysis and priorisation strategy. In addition DGC provided the COPD specific co-morbidity networks and PA implemented the pathway clustering method. JR conceived the integrative biomedical research platform and co-defined the user interface specification. MC provided input to the semantic mapping of mathematical models and the prioritisation validation. DM conceived, designed and specified the COPDKB, co-developed the architecture design and drafted the manuscript.

All authors read and approved the final manuscript.

## Ethics and informed consent

Ethics and informed consent for the clinical studies integrated within the COPDKB were obtained within the framework of the original studies. The COPDKB contains de-identified data and therefore requires no separate ethics and informed consent.

## Supplementary Material

Additional file 1 **Supplement_S1_user-manual**.pdf. Detailed description and graphical views of all functions available in the COPD knowledge base. Click here for file

## Declaration

The research described in this paper is partly and the publication charge fully supported by the Synergy-COPD European project (FP7-ICT-270086). The opinions expressed in this paper are those of the authors and are not necessarily those of Synergy-COPD project's partners or the European Commission.

This article has been published as part of *Journal of Translational Medicine *Volume 12 Supplement 2, 2014: Systems medicine in chronic diseases: COPD as a use case. The full contents of the supplement are available online at http://www.translational-medicine.com/supplements/12/S2.
