# Supplementary material for: The COPD Knowledge Base: enabling data analysis and computational simulation in translational COPD research
Source: J Transl Med. 2014 Nov 28;12(Suppl 2):S6. doi: 10.1186/1479-5876-12-S2-S6 (PMC4255911; doi:10.1186/1479-5876-12-S2-S6)
Supplement: Additional file 1 — Supplement_S1_user-manual.pdf. Detailed description and graphical views of all functions available in the COPD knowledge base. [file 1479-5876-12-S2-S6-S1.pdf]

COPDKB user manual

1.1 CLINICAL STUDY DATA 3
1.2 ANALYSIS 16
1.3 MODELS 21
1.4 PUBLIC KNOWLEDGE 25
1.5 DOCUMENTATION 32

COPD Knowledge base user manual

To give an overview of the functionality of the Portal, we provide a short, screenshot based guide on the information available within the COPD Knowledge base and the usage of the user interface.

Step 1: access the login page to access the secure, authentification and authorisation based COPDKB (Figure 1). Please not, due to inclusion of clinical data access requires registration at COPDKB@clinic.ub.es.

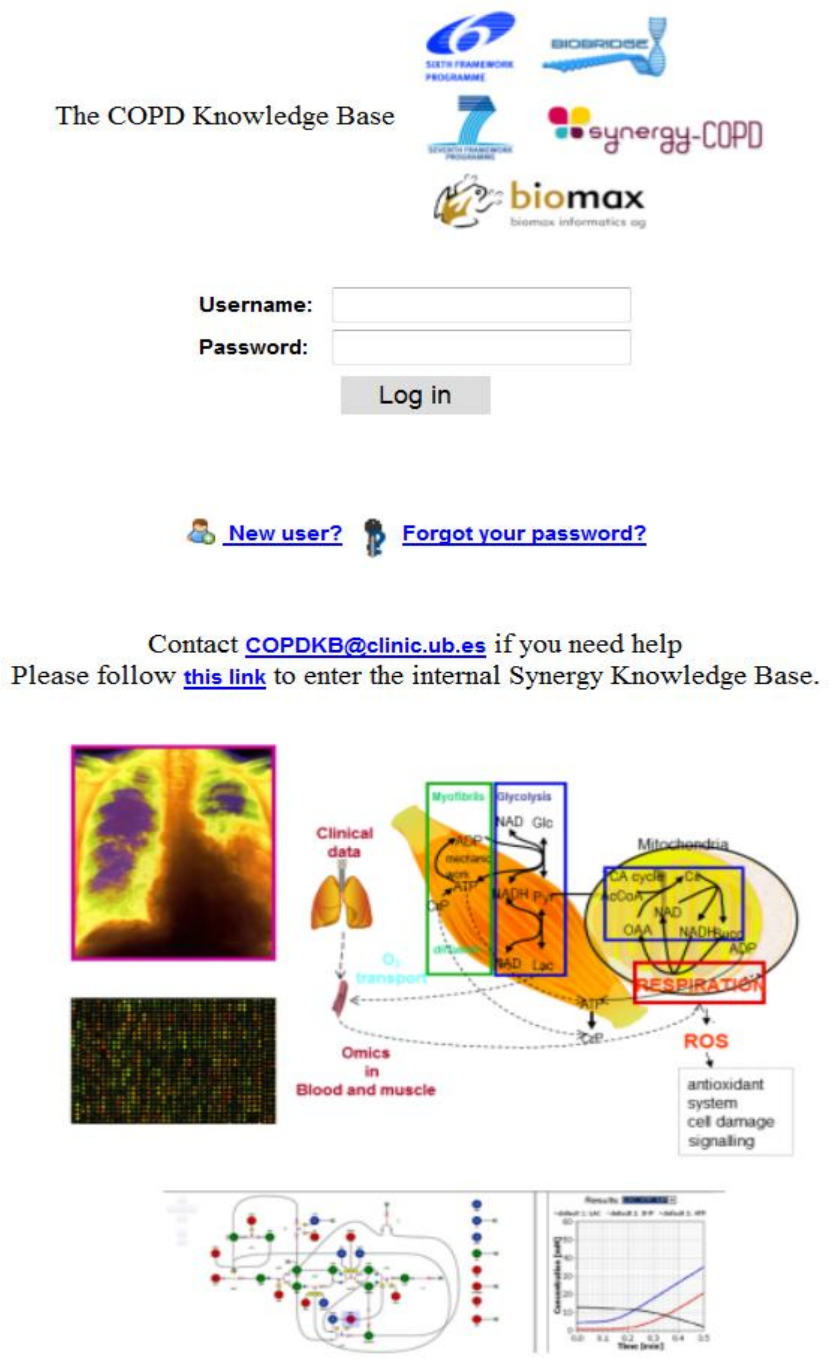

Figure 1 Login for the COPD Knowledge Base portal

Step 2: You are now on the Home page (Figure 2). On the left you can find a frame providing navigation to the different types of information integrated into the COPDKB.

On the home page main panel you can find a diagram providing an overview of the Synergy-COPD project structure, overall information becoming availbale during the project run-time and workflow (see below).

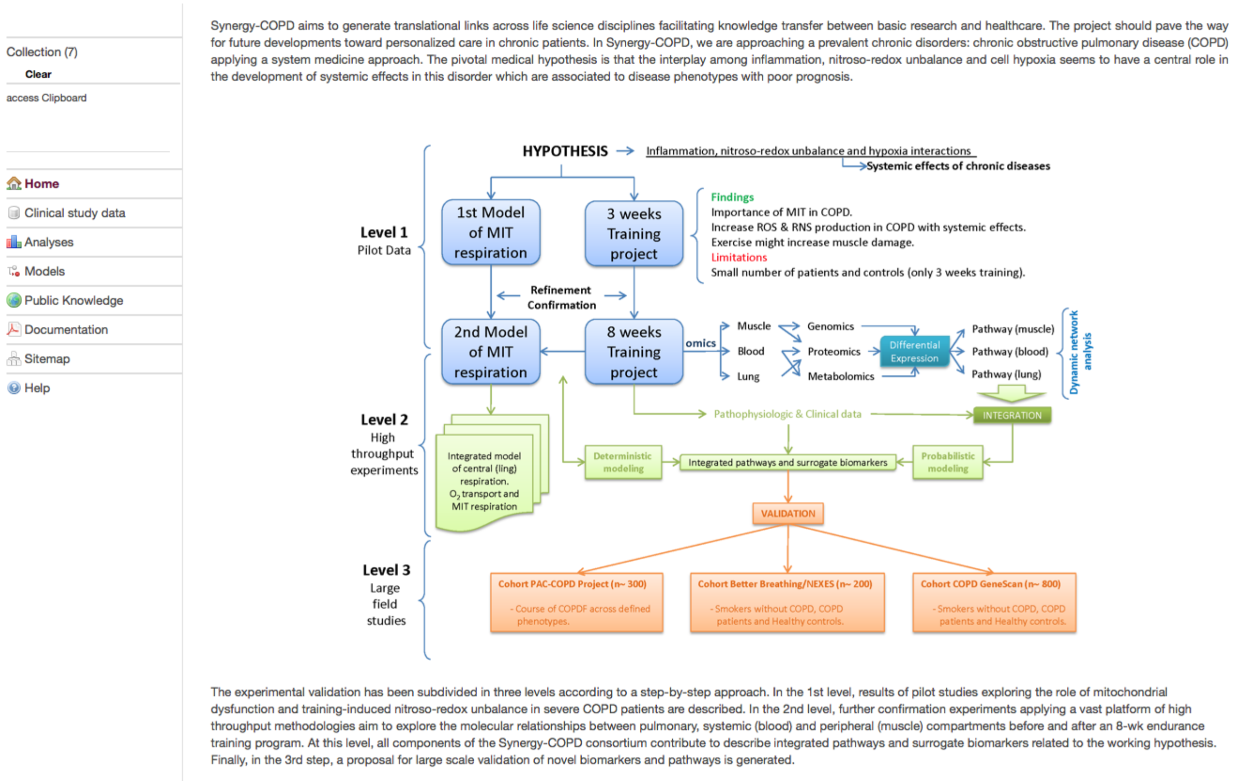

Figure 2 Knowledge portal

1.1 CLINICAL STUDY DATA

Figure 3 refers to the Clinical Study Data section, which provides access to the clinical patient and experimental measurement database integrated into the COPDKB.

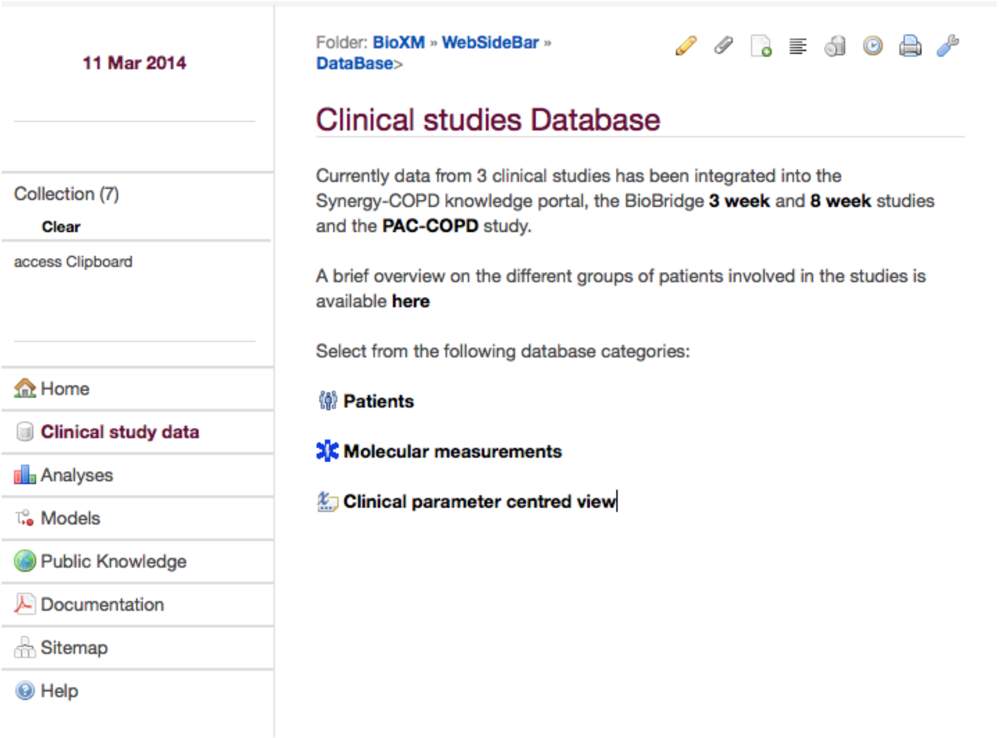

Figure 3 Clinical studies database

In this section you can find the Patient's data, an overview of patient related clinical data available in the COPDKB. Currently data from three clinical studies have been integrated into the COPD knowledge portal, the Biobridge 3-week and 8-week studies as well as the PAC-COPD study.

Step 1 Clicking on "Patients" brings you to the Biobridge and PAC-COPD patients browser where you can find pre-defined queries which allow to access all or specific subsets of clinical data. The patients browser for the Biobridge data is located directly on this page, whereas the browser for the PAC-COPD patients can be accessed by following the link "Filter PAC-COPD patients" at the top of the page (Figure 4).

BioBridge Patient Data: The BioBridge study was designed as a pilot study with a set of experimental studies aimed to test the hypothesis that mitochondrial alterations and nitroso-redox unbalance are centrally involved in skeletal muscle dysfunction and reduced exercise capacity in patients with COPD. According to these aims, a 3-week and an 8-week Training Project were designed to obtain the appropriate clinical, functional and biological information.
In both training projects groups of patients with COPD and healthy controls were studied in two conditions:

- At rest before training (BT)
- At rest after training (AT)

Measurements of relevant clinical parameters, respiratory function and exercise capacity were performed as shown in Figure 4. In addition, open biopsies from the vastus lateralis (quadriceps) were obtained before training and at the post-training time point. The following experimental studies from muscle tissue were conducted:

- Myofiber respiratory mitochondrial function.
- Oxidative and nitrosative stress-induced muscle protein modifications.
- Muscle transcriptomics by microarray analysis.

PAC-COPD Patients browser

- **Filter PAC-COPD patients**

Biobridge Patients browser

BMI [Consider all] Training [Consider all] [Filter Patients]

Alternatively you can select one of the following pre-defined queries:

[View All Patients] [Execute Query]

Overview

We designated as pilot studies a set of experimental studies aimed to test the hypothesis that mitochondrial alterations and nitroso-redox unbalance are centrally involved in skeletal muscle dysfunction and reduced exercise capacity in patients with COPD. According to these aims, a 3-week and a 8-week Training Project were designed to obtain the appropriate clinical, functional and biological information. In the both Training Project groups of patients with COPD and healthy controls were studied in two conditions:

- At rest before training (BT)
- At rest after training (AT)

Measurements of relevant clinical parameters, respiratory function and exercise capacity were performed. In addition, open biopsies from the vastus lateralis (quadriceps) were obtained in all participants before training and in all but 2 COPD patients and 2 healthy subjects, who refused a second muscle biopsy, at the post-training time point. The following experimental studies from muscle tissue were conducted:

- Myofiber respiratory mitochondrial function.
- Oxidative and nitrosative stress-induced muscle protein modifications.
- Muscle transcriptomics by microarray analysis.

Study population

Fifteen/thirtyone (3-wk/8-wk) patients with stable severe COPD and 7/12 healthy age-matched sedentary controls were recruited. The COPD group was sub-classified as low BMI (≤ 21 kg/m2, BMIL) (n= 7/6) and normal BMI (>21 kg/m2, BMIN) (n= 8/13). Anthropometric and functional characteristics of the subjects at baseline according to Fat Free Mass Index (FFMI) are described in the following Table for the 3-wk study:

	Control Subjects n= 7	COPD Patients n= 15	$FFMI_N$ COPD patients n= 8	$FFMI_L$ COPD patients n= 7
Age, yrs	62 (6)	62 (7)	63 (6)	61 (9)
BMI, Kg /m²	27.9 (5.1)	24.4 (6.2)	29.0 (4.8)	19.2 (1.7) §§§, †††
FFMI, Kg /m²	22.3 (2.5)	20.0 (3.5)	22.6 (2.4)	17.0 (1.2) §§§, ††
FEV_1, % pred.	95 (15)	41 (18) ***	47 (19) §§§	33 (16) §§§
FVC, % pred.	97 (12)	68 (18) ***	80 (14) §	55 (13) §§§, ††
FEV_1/FVC, %	78 (5)	44 (12) ***	44 (12)	44 (12)
DLco, % pred.	N.A.	50 (21)	60 (18)	39 (8) ††
PaO_2, mmHg	95 (13)	75 (10) *	77 (14) §	73 (4) §§
$PaCO_2$, mmHg	37.4 (3.8)	42 (3.1)	42.5 (3.4)	41.7 (3.2)
VO_2peak, % pred	100 (16)	57 (30) *	77 (18) §	27 (4) §§§, †††

Statistical significance is expressed as follows: * p < 0.05, * p < 0.001, between COPD patients and control subjects; § p < 0.05, §§ p < 0.01, §§§ p < 0.001 between either FFMIN or FFMIL COPD patients and control subjects; † p < 0.05, †† p < 0.01, ††† p < 0.001 between FFMI_L and FFMI_N COPD patients.

Figure 4 Patients browser

All patients of a **selected sub-group** are presented in a list report. In Figure 5 age, gender and associated experimental data are shown for each patient. The “Search” option allows to filter the list e.g. for gender (image below).

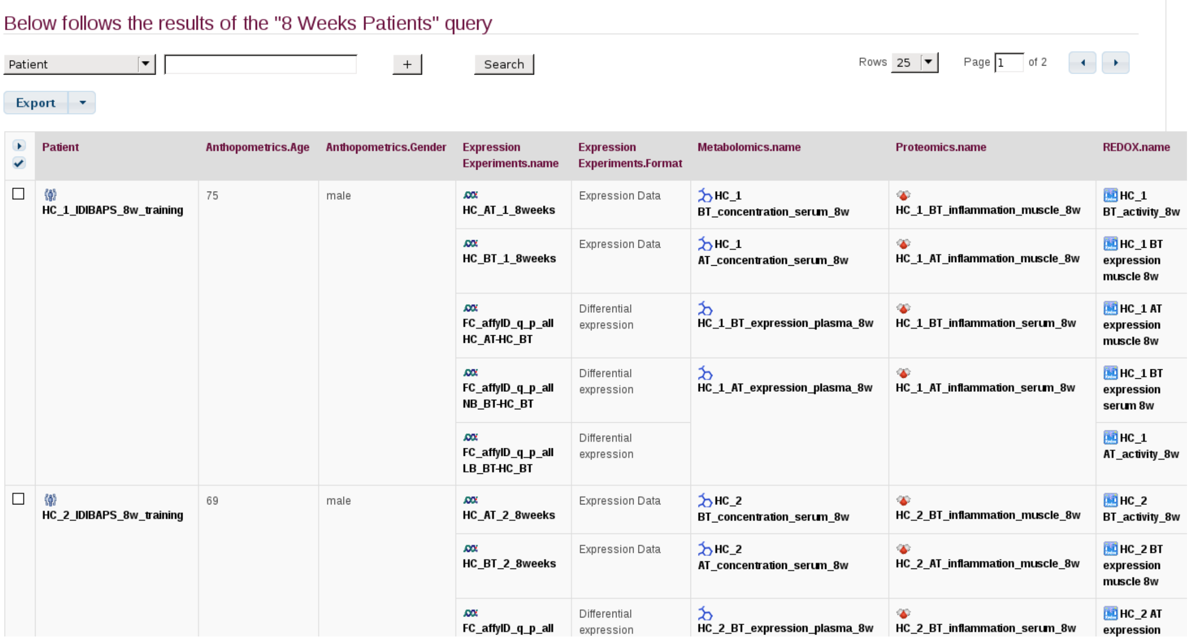

Below follows the results of the "8 Weeks Patients" query

Patient	Anthopometrics.Age	Anthopometrics.Gender	Expression Experiments.name	Expression Experiments.Format	Metabolomics.name	Proteomics.name	REDOX.name
HC_1_IDIBAPS_8w_training	75	male	HC_AT_1_8weeks	Expression Data	HC_1 BT_concentration_serum_8w	HC_1_BT_inflammation_muscle_8w	HC_1 BT_activity_8w
			HC_BT_1_8weeks	Expression Data	HC_1 AT_concentration_serum_8w	HC_1_AT_inflammation_muscle_8w	HC_1 BT expression muscle 8w
			FC_affyID_q_p_all HC_AT-HC_BT	Differential expression	HC_1_BT_expression_plasma_8w	HC_1_BT_inflammation_serum_8w	HC_1 AT expression muscle 8w
			FC_affyID_q_p_all NB_BT-HC_BT	Differential expression	HC_1_AT_expression_plasma_8w	HC_1_AT_inflammation_serum_8w	HC_1 BT expression serum 8w
			FC_affyID_q_p_all LB_BT-HC_BT	Differential expression			HC_1 AT_activity_8w
HC_2_IDIBAPS_8w_training	69	male	HC_AT_2_8weeks	Expression Data	HC_2 BT_concentration_serum_8w	HC_2_BT_inflammation_muscle_8w	HC_2 BT_activity_8w
			HC_BT_2_8weeks	Expression Data	HC_2 AT_concentration_serum_8w	HC_2_AT_inflammation_muscle_8w	HC_2 BT expression muscle 8w
			FC_affyID_q_p_all	Differential expression	HC_2_BT_expression_plasma_8w	HC_2_BT_inflammation_serum_8w	HC_2 AT expression

Figure 5 Patient's follow up

Individual patient reports are available upon click on the patient name (Figure 6). Again multiple reports providing different types of information can be selected from the left frame (here “Patient clinical data (WIKI)” schown in bold in the left frame).

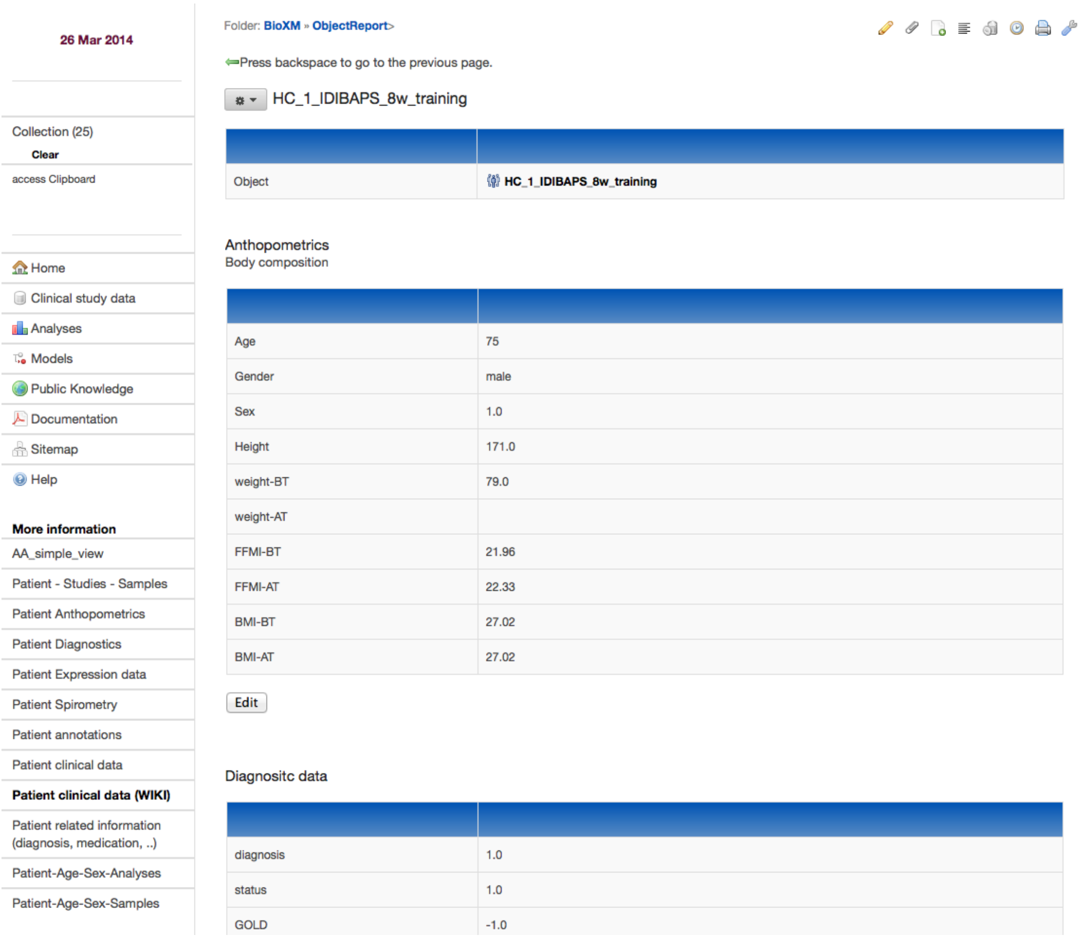

Figure 6 Patient's individual report

PAC-COPD Patient Data: Data for this patient group can be retrieved in a different way by following the link “Filter PAC-COPD patients” at the top of the patient browser page (Figure 4). On the following page one can filter all PAC-COPD patients by parameter, e.g. age (Figure 7). Please note, the threshold boxes have to be filled in in any case and all patients are reported where at least one selected parameter falls within the thresholds.

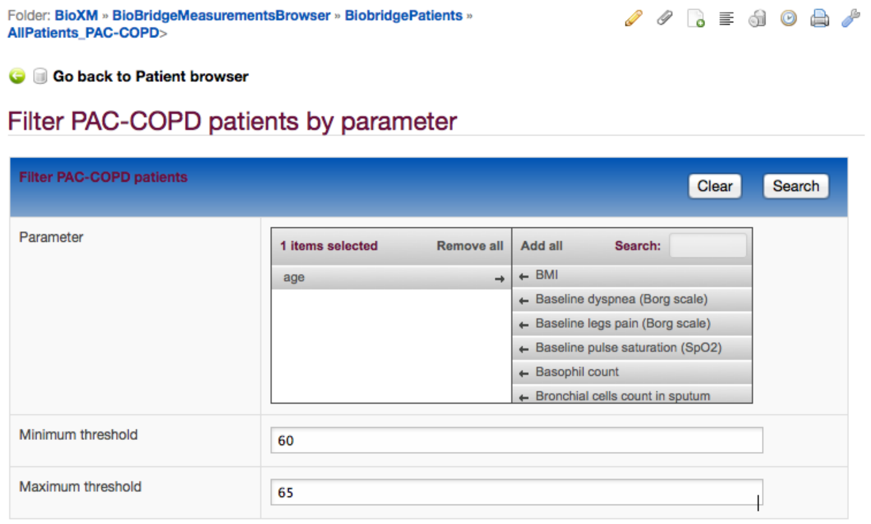

Figure 7 Patient selection by parameter

Upon selection of patients on the result page (not shown) with check marks, the data matrix of these patients can be displayed by clicking “Clinic data” in the drop-down menu “Data matrix”. The resulting table shows the values of all measured parameters for these patients (Figure 8).

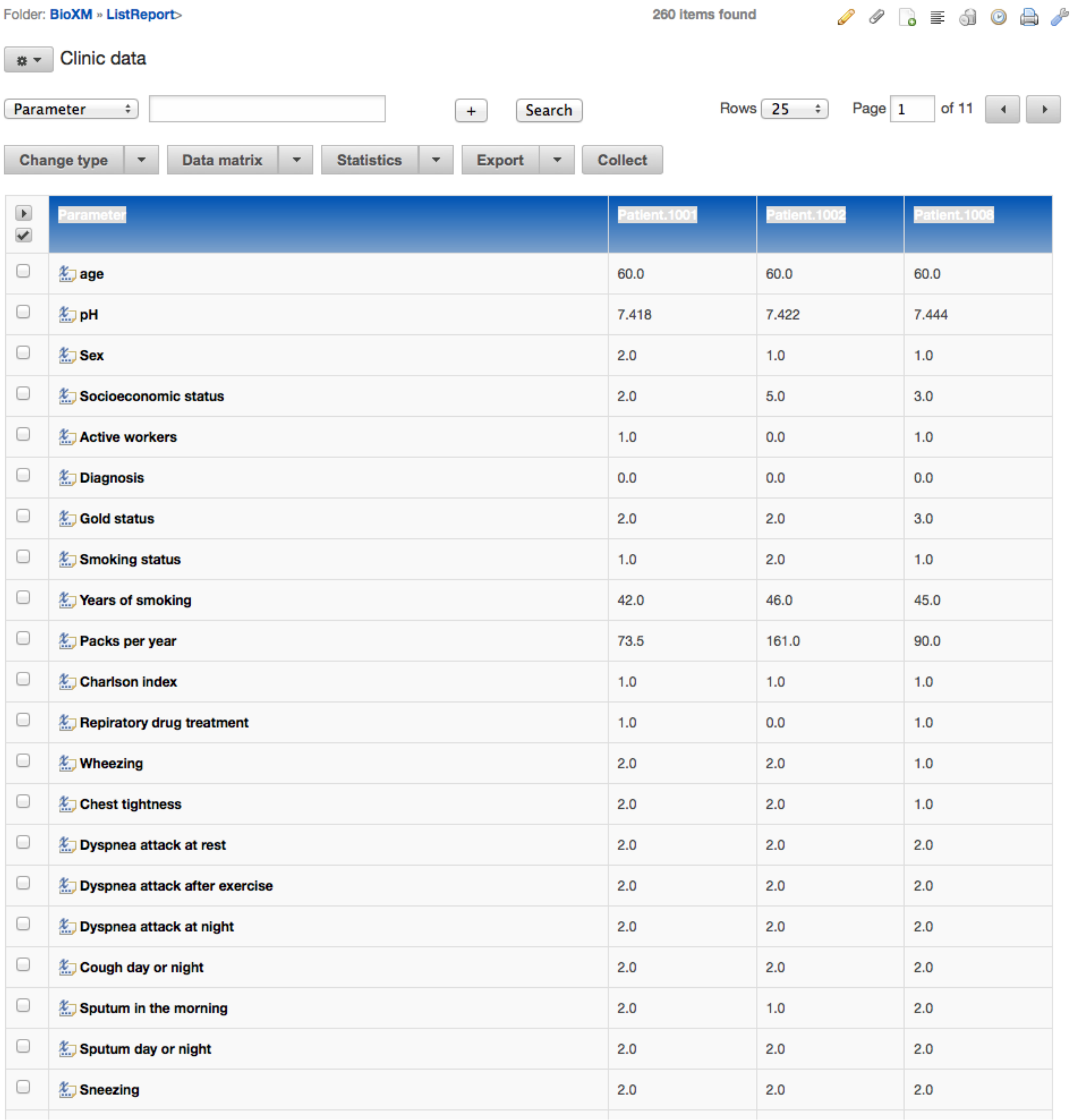

Parameter	Patient.1001	Patient.1002	Patient.1008
age	60.0	60.0	60.0
pH	7.418	7.422	7.444
Sex	2.0	1.0	1.0
Socioeconomic status	2.0	5.0	3.0
Active workers	1.0	0.0	1.0
Diagnosis	0.0	0.0	0.0
Gold status	2.0	2.0	3.0
Smoking status	1.0	2.0	1.0
Years of smoking	42.0	46.0	45.0
Packs per year	73.5	161.0	90.0
Charlson index	1.0	1.0	1.0
Repiratory drug treatment	1.0	0.0	1.0
Wheezing	2.0	2.0	1.0
Chest tightness	2.0	2.0	1.0
Dyspnea attack at rest	2.0	2.0	2.0
Dyspnea attack after exercise	2.0	2.0	2.0
Dyspnea attack at night	2.0	2.0	2.0
Cough day or night	2.0	2.0	2.0
Sputum in the morning	2.0	1.0	2.0
Sputum day or night	2.0	2.0	2.0
Sneezing	2.0	2.0	2.0

Figure 8 Data matrix of selected patients

The buttons on the top of the table provide methods to filter or futher extend from the clinical data to molecular measurements, diseases or simple statistics

Statistics – provides simple summary statistics as well as two group comparisons. Select clinical parameters of interest and after clicking the “Statistics” button select the patients for group one and two. “Summary” will aggregate all selected patients, “Comparison” will provide a t-test between the two groups for each individual parameter (see Figure 9).

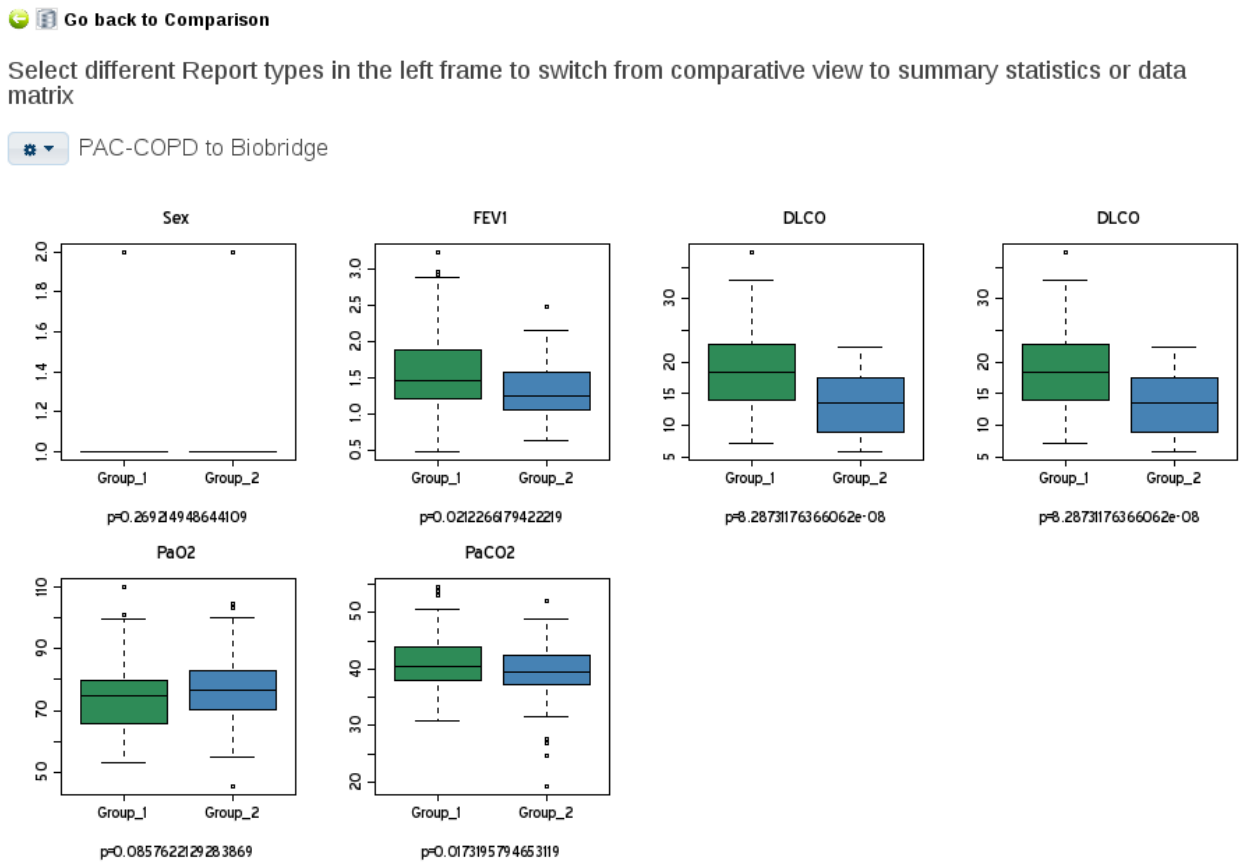

Figure 9 T-test based comparative statistics for 6 selected clinical attributes and the patients selected into group 1 and 2

Step 2 Selecting **"Molecular measurements"** (Figure 3) in the "Clinical study data" section brings the user to the "Biobridge studies molecular data browser" (Figure 10) where there is an overview of currently available experimental data *(restricted to the BioBridge study).*

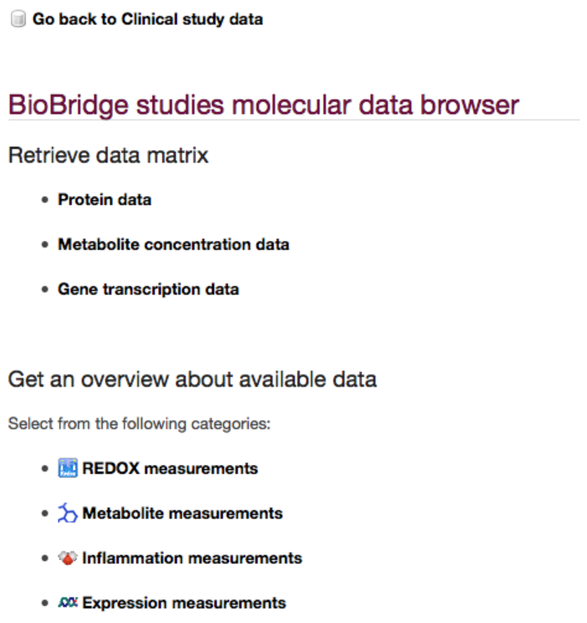

Figure 10 Overview of different menues offered by BioBridge database

Example Figure 11 shows the "Inflammation Measurements Browser": selecting all measurements of inflammation markers corresponding to the **filter criteria "BMI=Low", "State=After training"**

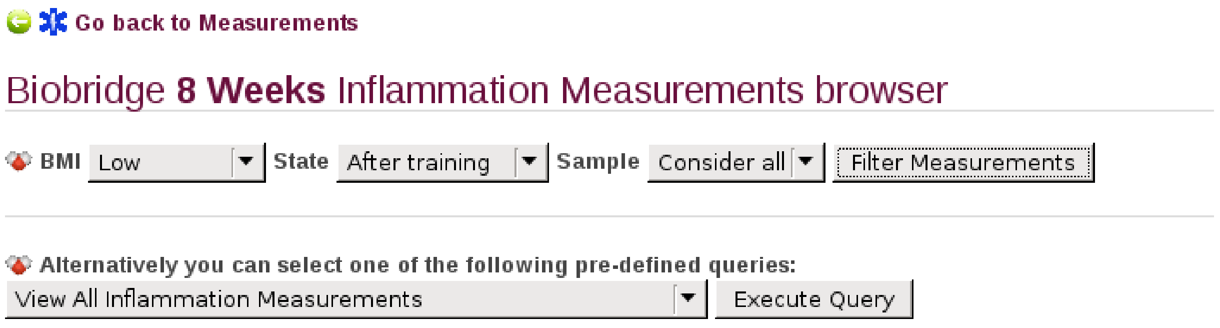

Figure 11 Inflammation measurements browser

If you wish you can also select a view of the **List of all available inflammation measurements** shown in Figure 12. Clicking on table headers sorts the table, "Search" filters by selected table column (multi-column filtering available).

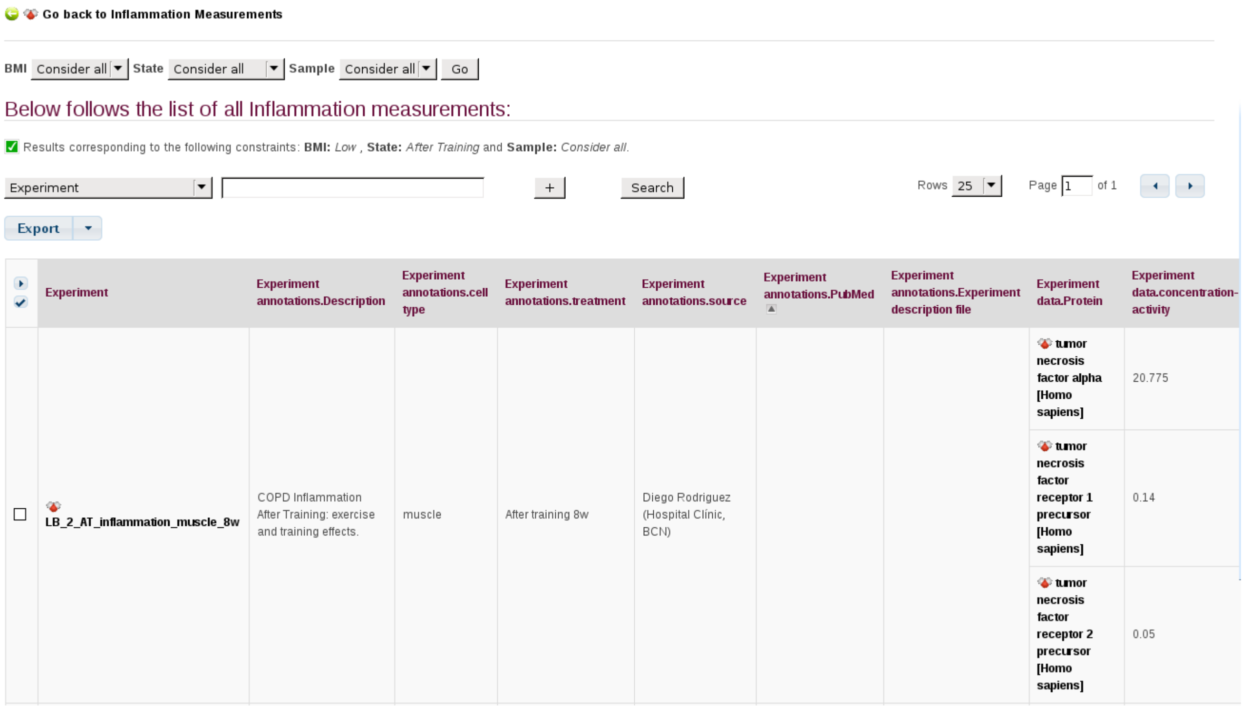

Figure 12 List of inflammation measurements

By clicking on the experiment name and selecting the view "Experiment – Experiment data" in the left panel, you obtain details about an Inflammation marker measurement as presented in Figure 13. Experimental conditions, measured marker, measured value.

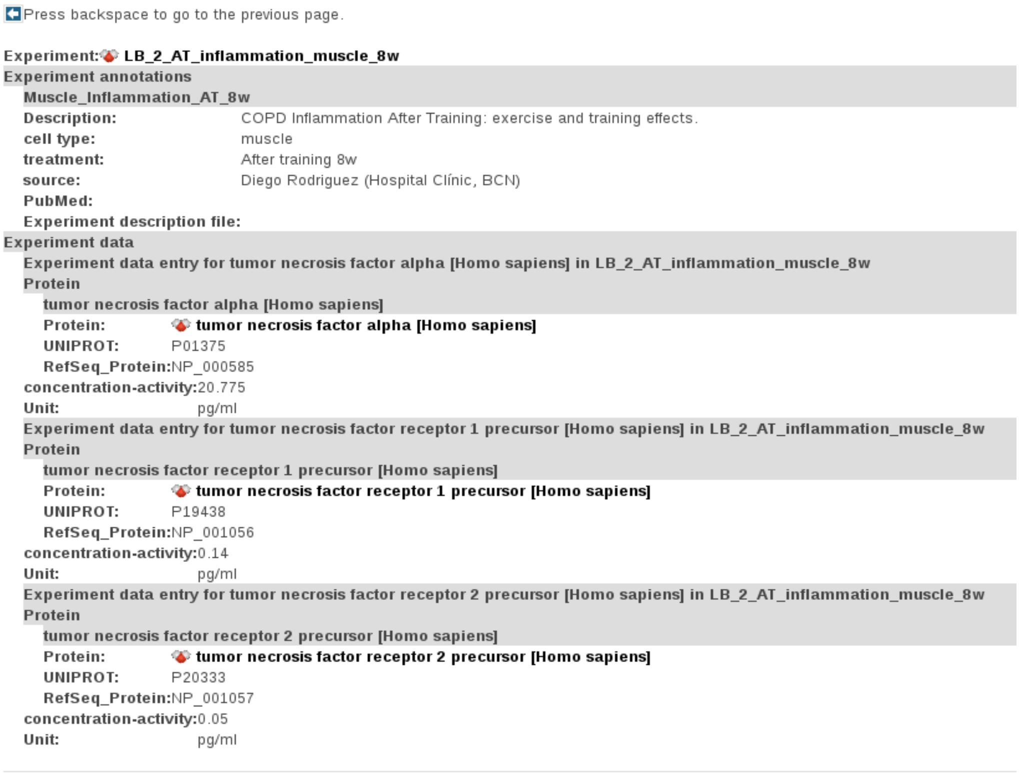

Figure 13 Inflammation measurement details

Step 3 Selecting **"Clinical parameter centered view"** in the "Clinical study data" section (Figure 3) brings the user to a list of predefined queries selecting all or certain sets of clinical parameters measured in the PAC-COPD and BioBridge studies (Figure 14).

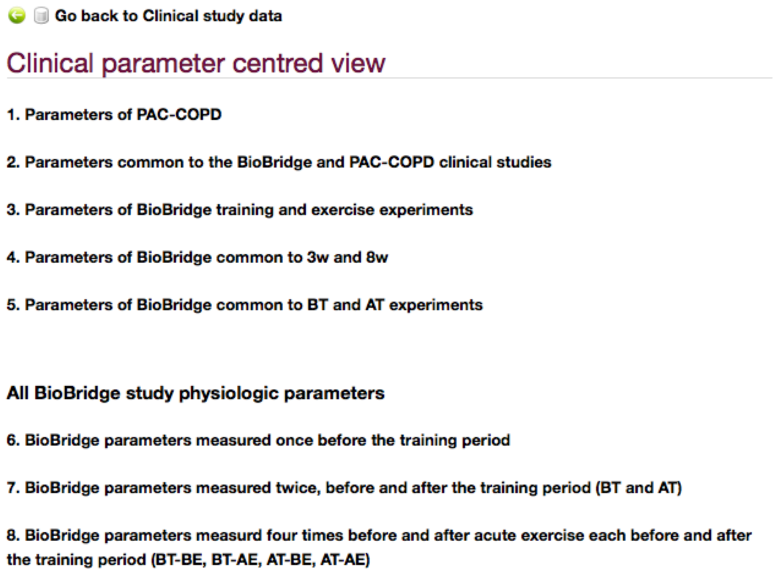

Figure 14 Clinical parameter centered view

Example The query "2. Parameters common to the BioBridge and PAC-COPD clinical studies" selects all parameters that were measured in both studies and displays them along with parameter specific information in a result table ("Parameter report view", Figure 15). An alternative "Data matrix view" for these parameters can also be selected and results in a data matrix reporting the values for all common parameters measured in all BioBridge and PAC-COPD patients (Figure 16).

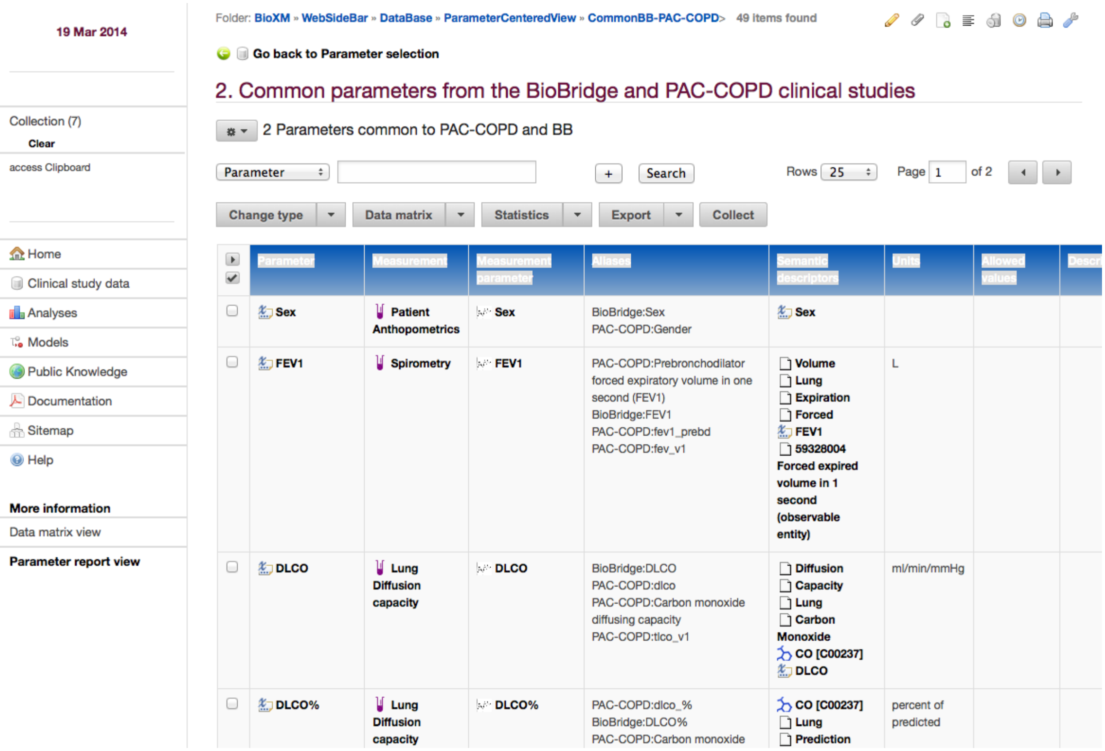

Figure 15 Parameter report view

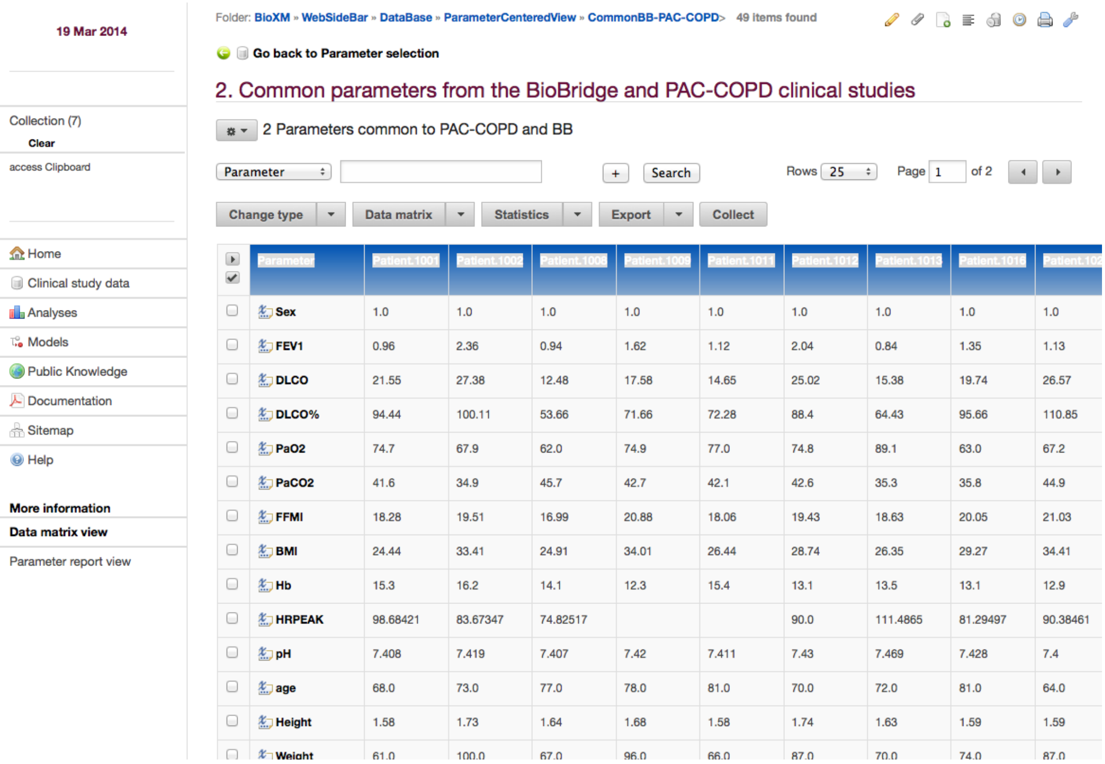

Figure 16 Data matrix view

To display the data matrix of specific parameters and specific patients, first the parameters have to be selected with check marks and the appropriate data matrix has to be chosen from the drop-down menu

“Data matrix” (here “2 Parameters common to PAC-COPD and BB”). In a next step patients can be chosen (Figure 17) and the data matrix displayed (Figure 18).

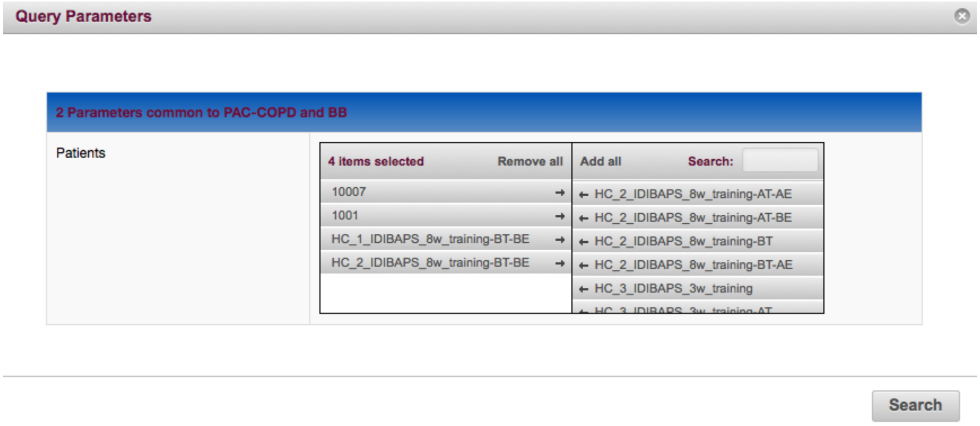

Figure 17 Patient selection

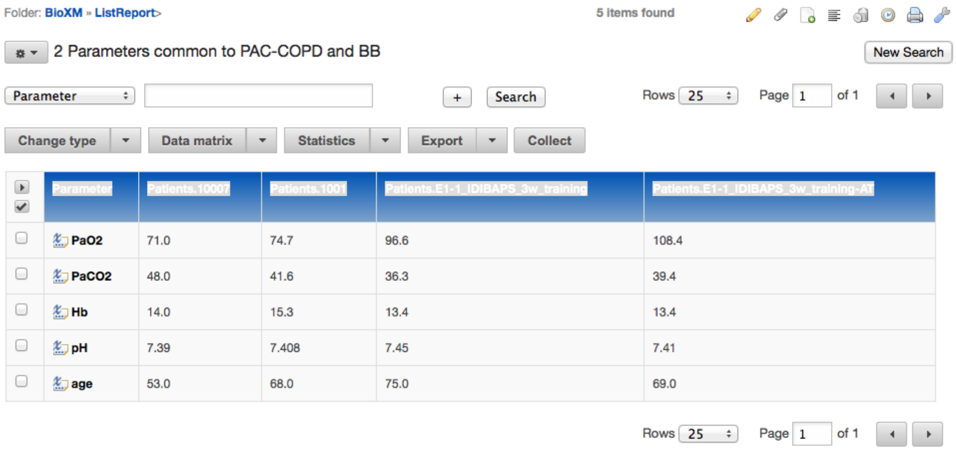

Parameter	Patients.10007	Patients.1001	Patients.E1-1_IDIBAPS_3w_training	Patients.E1-1_IDIBAPS_3w_training-AT
PaO2	71.0	74.7	96.6	108.4
PaCO2	48.0	41.6	36.3	39.4
Hb	14.0	15.3	13.4	13.4
pH	7.39	7.408	7.45	7.41
age	53.0	68.0	75.0	69.0

Figure 18 Data matrix for selected parameter and patients from BioBridge and PAC-COPD studies

1.2 ANALYSIS

The “Analysis” section provides access to available analysis results. Currently sequence similarity based gene homology, differentíal expression analysis and probabilistic networks are available in the browser portal (Figure 19).

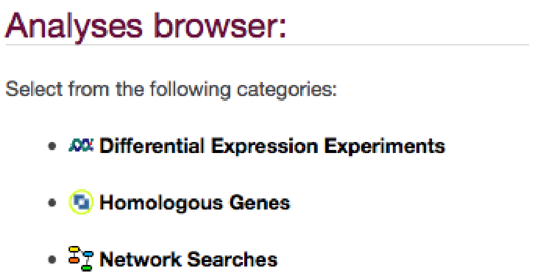

Figure 19 Analysis tab overview

Fold-change based comparisons are available for **healthy - COPD, normal - low BMI, before - after training.** (Figure 20)

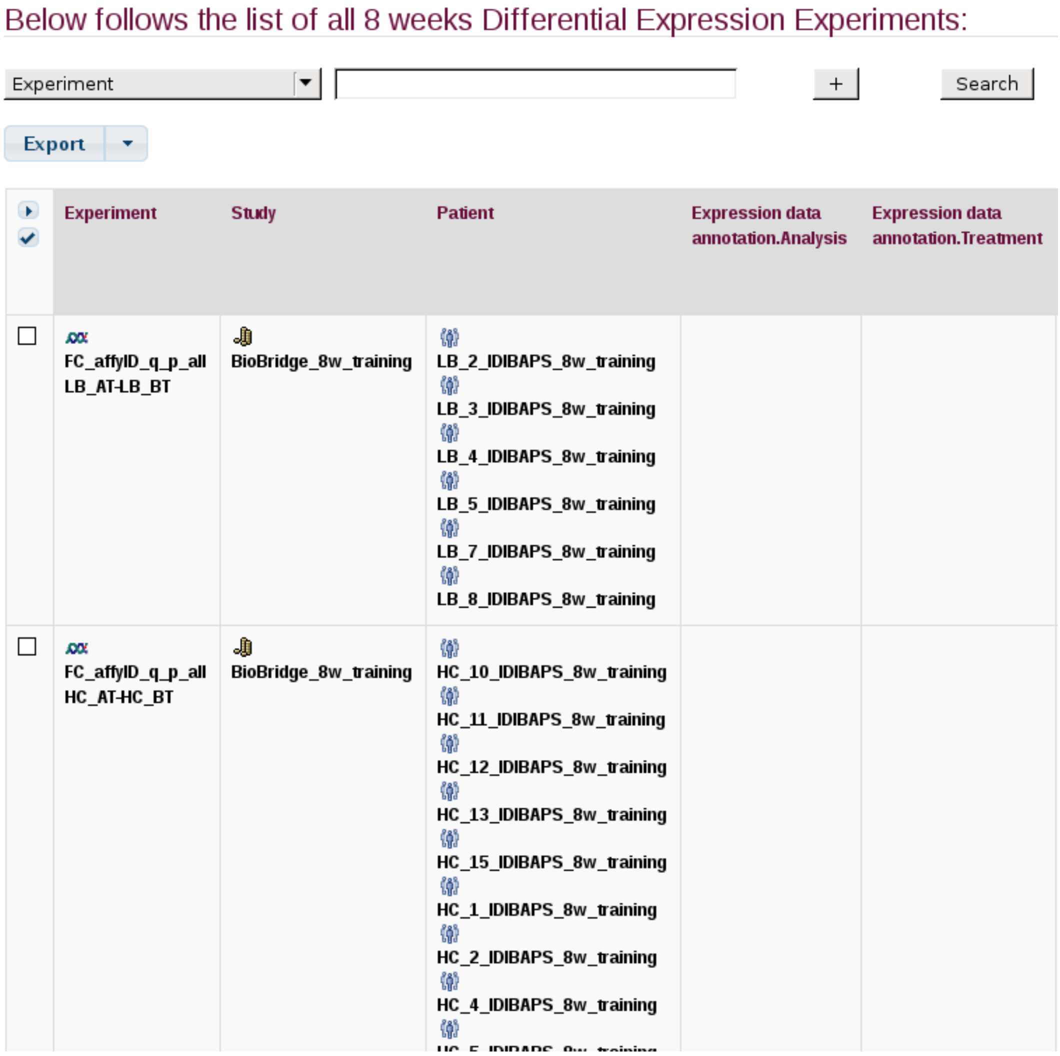

Figure 20 Differential expression experiments

Detailed descriptions are provided as part of the individual analysis report: upon click on an analysis name in the overview table and slection of the view “Experiment – Study – Patients” in the left panel as shows Figure 21.

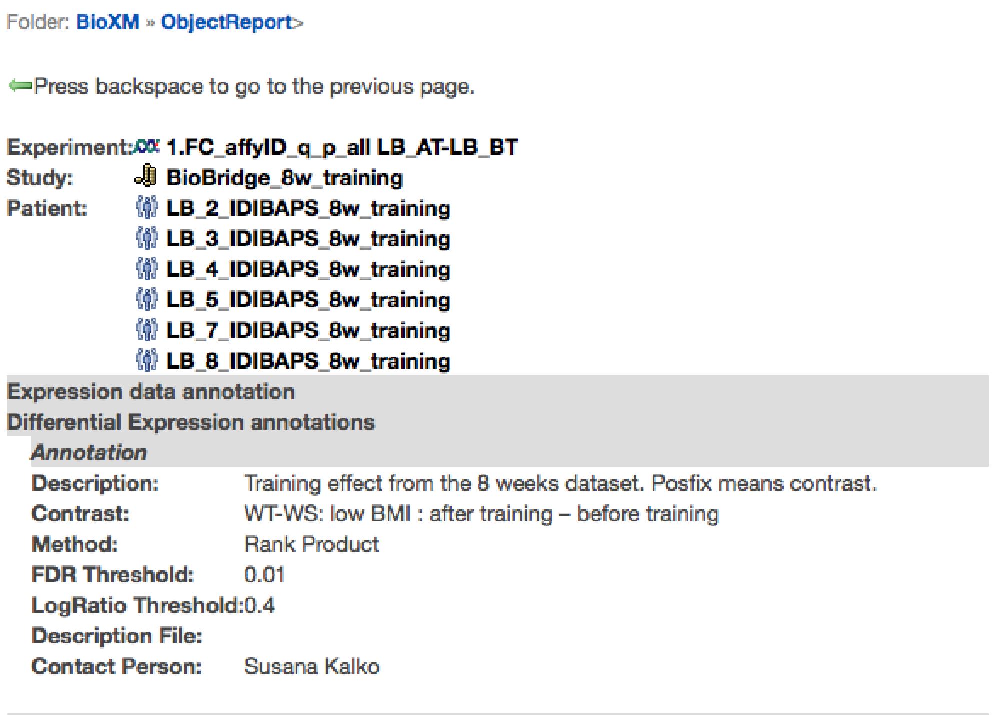

Figure 21 Description of individual analysis

The **“Network Searches”** section allows to search for all defined connection types that join a set of objects by a maximum of x intermediate steps. One example would be to select a list of genes e.g. from the differential expression analysis and then check whether these are somehow functional connected by searching for protein-protein interactions, gene regulation interaction, metabolic reactions and association within the same signalling pathway which will connect any of the selected genes by a maximum of 8 intermediate steps.

The first block on the “Network Searches” page lists networks based on protein-protein interactions whereas the second block give access to a set of precalculated protein networks based on overrepresentation analyses (Figure 22).

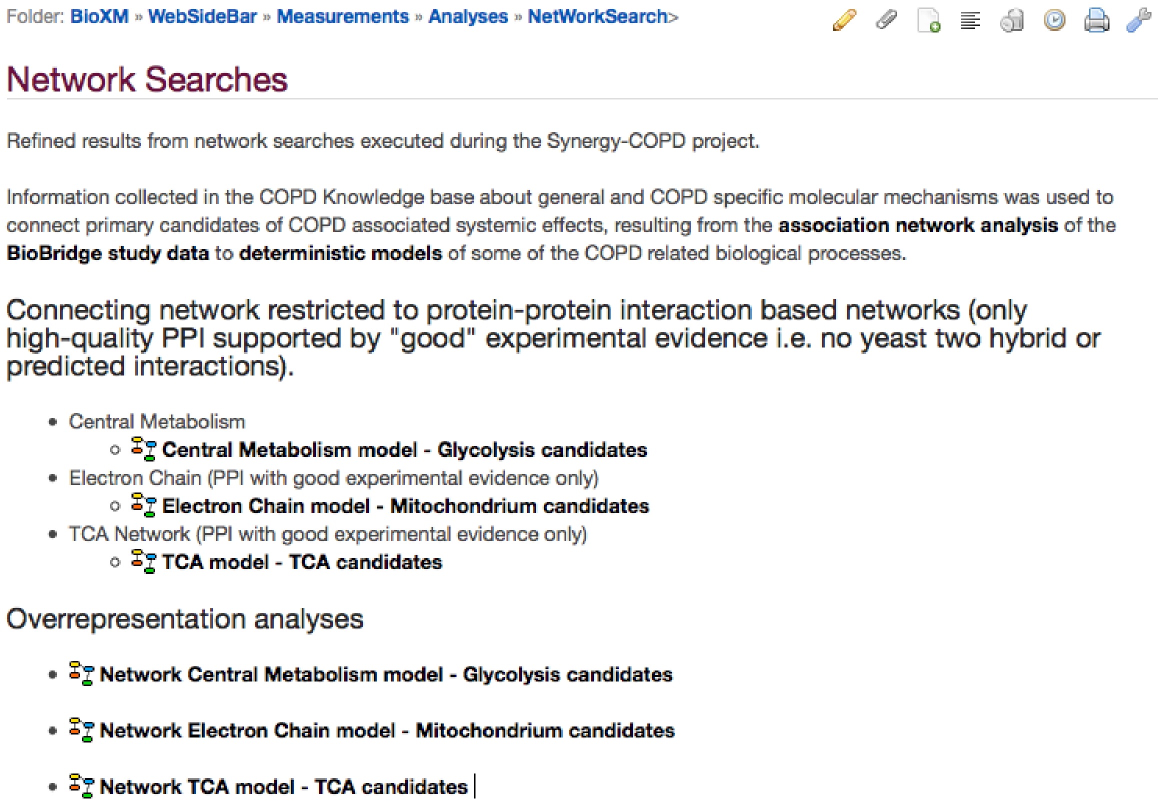
Folder: BioXM » WebSideBar » Measurements » Analyses » NetWorkSearch>

Network Searches

Refined results from network searches executed during the Synergy-COPD project.

Information collected in the COPD Knowledge base about general and COPD specific molecular mechanisms was used to connect primary candidates of COPD associated systemic effects, resulting from the **association network analysis** of the **BioBridge study data** to **deterministic models** of some of the COPD related biological processes.

Connecting network restricted to protein-protein interaction based networks (only high-quality PPI supported by "good" experimental evidence i.e. no yeast two hybrid or predicted interactions).

- Central Metabolism
  - **Central Metabolism model - Glycolysis candidates**
- Electron Chain (PPI with good experimental evidence only)
  - **Electron Chain model - Mitochondrium candidates**
- TCA Network (PPI with good experimental evidence only)
  - **TCA model - TCA candidates**

Overrepresentation analyses

- **Network Central Metabolism model - Glycolysis candidates**
- **Network Electron Chain model - Mitochondrium candidates**
- **Network TCA model - TCA candidates**

Figure 22 Network searches

Results of protein-protein interaction networks are presented as tables listing source and target proteins as well as their network associations as shown in (Figure 23). Click on a column header sorts the table. Click on the button next to the headline shows the graph of the interaction network, which also can be opened in a Java-based graph editor (Figure 24).

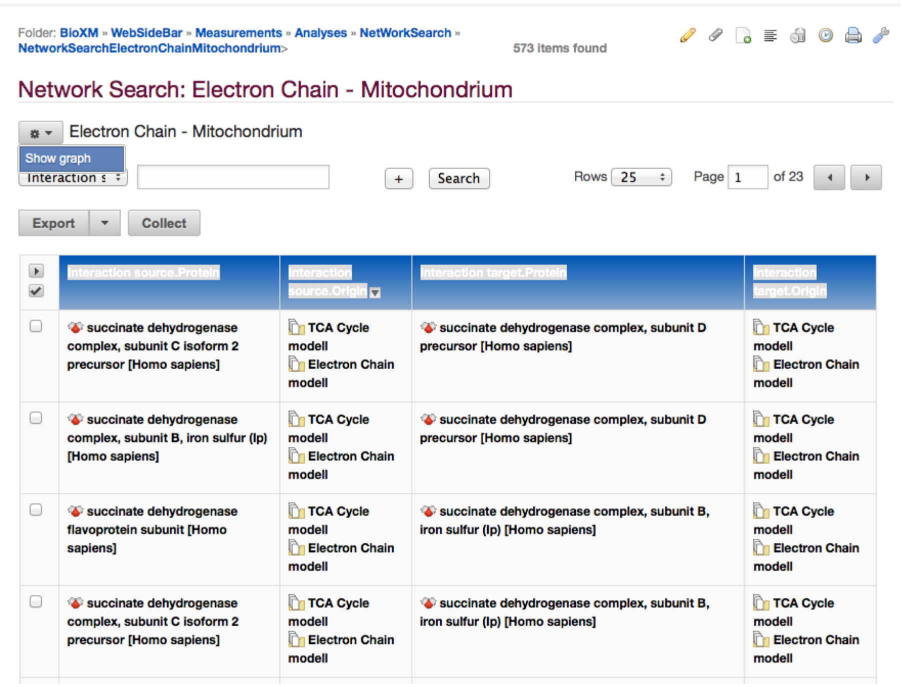

Figure 23 Protein-protein interaction table

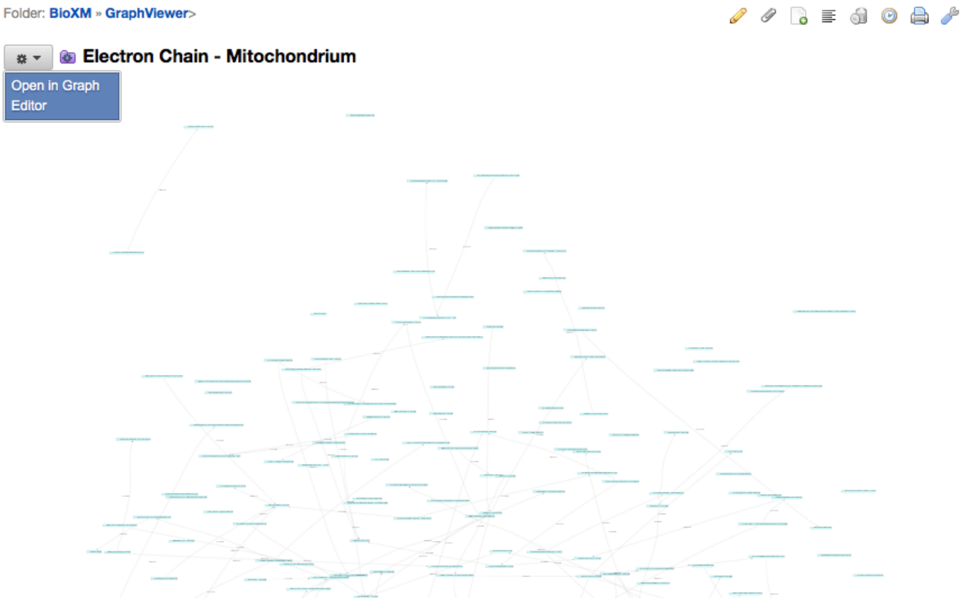

Figure 24 Protein-protein interaction graph

Results of overrepresentation networks are presented as table (Figure 25) where the genes of each individual pathway or sub-netwrk can be retieved (Figure 26).

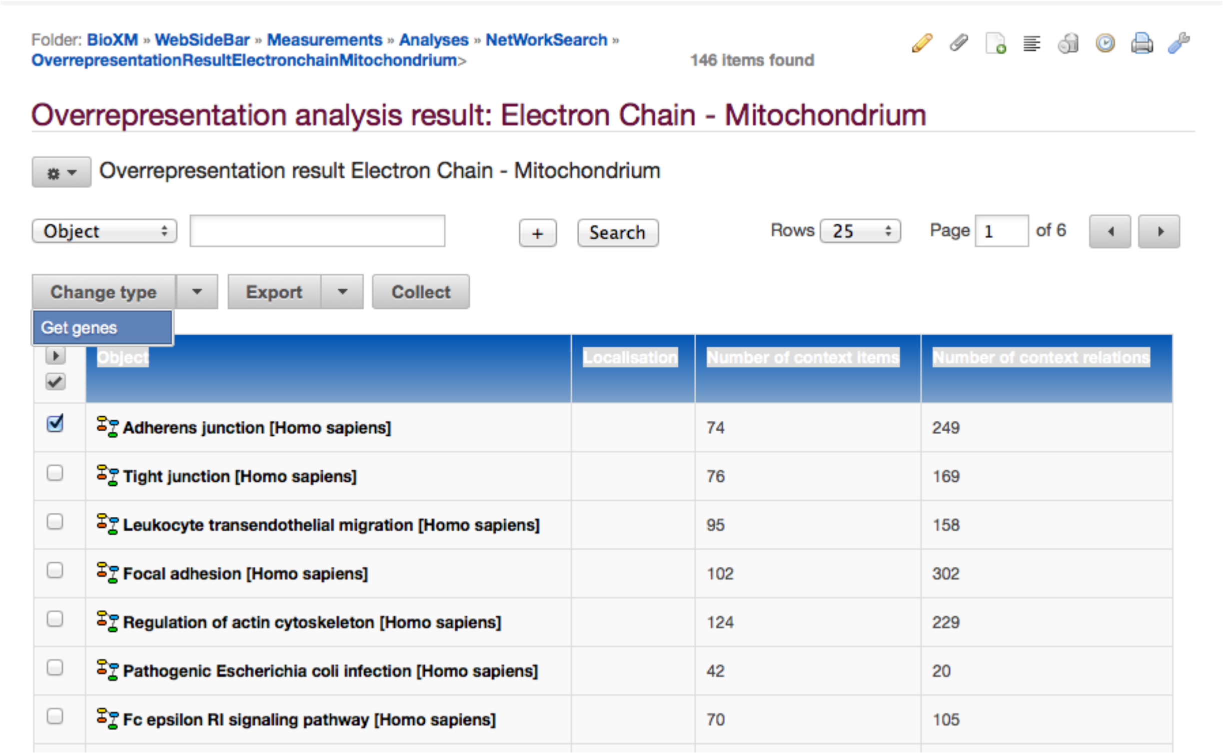

Figure 25 Overrepresentation network

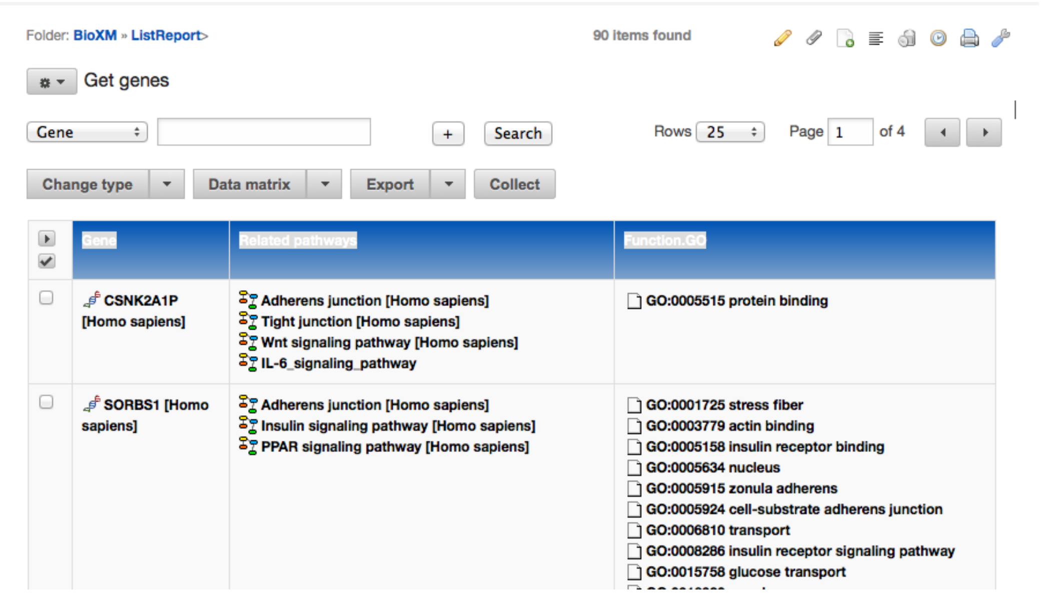

Figure 26 Genes in network

1.3 MODELS

In this section you can find the computational models being developed and integrated within the Synergy-COPD project.

There is an overview description available for each model, if you click on a model name you will be directed to it (Figure 27 and Figure 28).

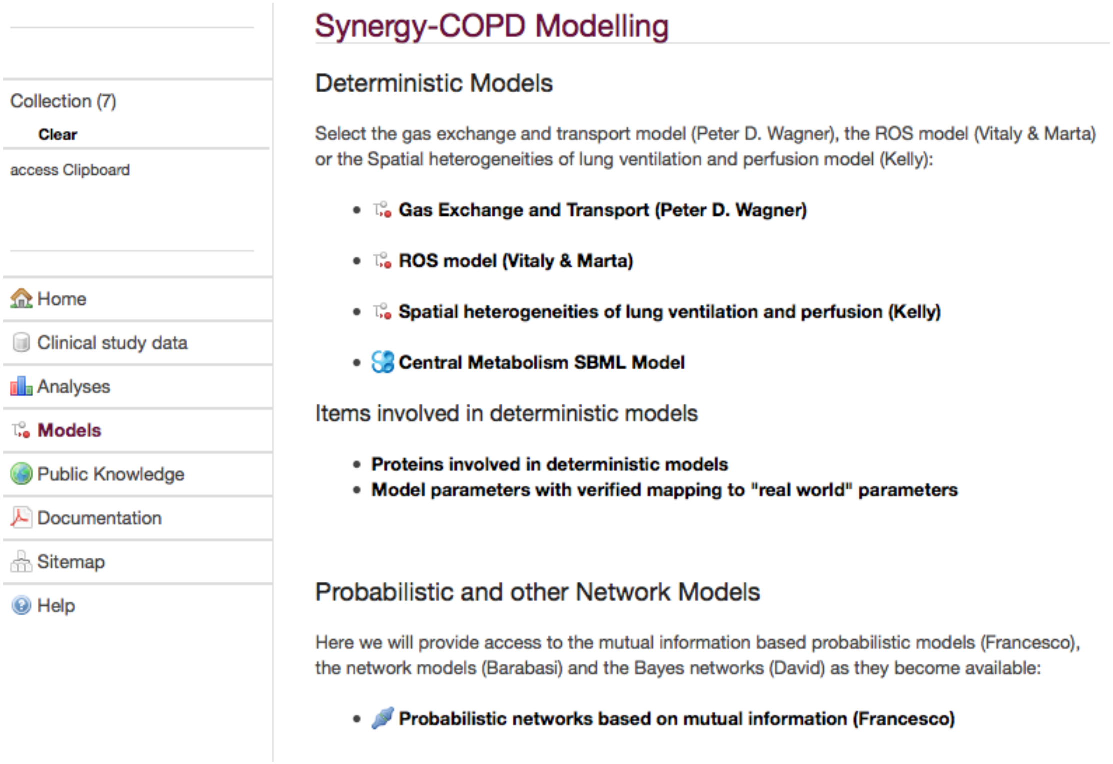

Figure 27 Overview of models tab

Gas exchange and Transport (Peter D.Wagner)

Go back to Models

Gas Exchange and transport Model annotation:

We have imported into the Synergy-COPD knowledge base (BioXM™) the *Gas Exchange and Transport* model annotations provided by Peter D. Wagner. The following is the description of the model. To go to the annotation of the model's parameters please right-click on the following link:

- **Go to the Gas Exchange and transport annotations**

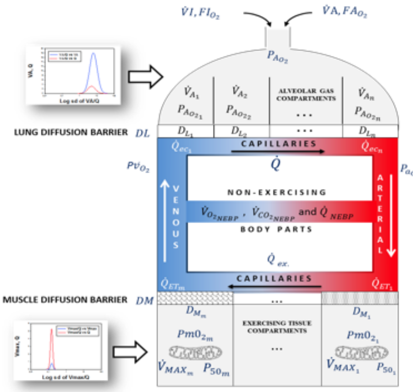

$$\dot V_{O_2} = \dot V I \cdot FI_{O_2} - \dot V A \cdot FA_{O_2} \quad \text{(a)}$$

$$\frac{d[O_2](t)}{dt} = \frac{DL}{T_L \cdot \dot Q} \cdot \left(PA_{O_2} - Pc_{O_2}(t)\right) \quad \text{(b)}$$

$$\dot V_{O_2} = \dot Q\left(Ca_{O_2} - C\bar v_{O_2}\right) \quad \text{(c)}$$

$$\frac{d[O_2](t)}{dt} = \frac{DM}{T_M \cdot \dot Q} \cdot \left(Pc_{O_2}(t) - Pm_{O_2}\right) \quad \text{(d)}$$

$$\dot V_{O_2} = \frac{\dot V_{MAX} \cdot Pm_{O_2}}{(Pm_{O_2} + P_{50})} \quad \text{(e)}$$

O2 transport is accomplished by 4 organs/tissues - lungs, heart, blood and muscles. Transport through this system involves four sequential processes or steps:

1. VENTILATION TO BRING O2 FROM AIR TO THE ALVEOLAR GAS: **(a)**
2. DIFFUSION OF O2 FROM ALVEOLAR GAS INTO CAPILLARY BLOOD: **(b)**
3. CIRCULATORY TRANSPORT OF O2 FROM LUNGS TO TISSUE MICROVESSELS: **(c)**
4. DIFFUSION OF O2 FROM TISSUE MICROVESSELS TO MITOCHONDRIA: **(d)**

Modelling is based on well-established principles of mass conservation for O2 at every step, using correspondingly well-established transport equations. There is one equation for each of the above 4 steps.

A fifth equation brings in mitochondrial metabolism. It expresses the rate of mitochondrial O2 consumption as an exponential, two parameter function of mitochondrial PO2. The two parameters are the P50 and VO2max of the exponential relationship:

5. MITOCHONDRIAL METABOLISM: **(e)**

Implicit in the above is knowing the HbO2 dissociation curve in a quantitative sense. This means that if we know PO2, we know [O2]. As should be clear, we have 5 equations and 5 unknowns. This means a unique solution can be obtained. This model finds the solution for desired set of input variables.

There are some important assumptions/approximations:

- O2 transport is in a steady state - all input and output variables are constant in time.

Figure 28 Gas exchange and transport model

In the overview description a link to the detailed semantic model and model parameter description is provided (Figure 29). The semantic description is used to map parameters between different models (see D3.1 Requirements Specification).

Press backspace to go to the previous page.

Gas Exchange and transport model annotations:

Model: **Gas exchange and transport**
Creation time: May 26, 2011 1:27:50 PM
Creation user name: admin
Last modification time: Jan 29, 2014 10:42:35 AM
Last modification user name: icanon
Model target:
- **GO:0007585 respiratory gaseous exchange**
- **GO:0008015 circulation**
- **FMA:7195 Lung**
- **FMA:7088 Heart**
- **FMA:30316 Muscle**

Execution information

Gas exchange and transport

Programming language: Java 1.7
Compiler version: windows system: JDK 1.7 or above.
Required libraries:
Supported operating systems: Windows
binaries: **Raw File (104 Kb) (model6.jar)**

Model semantics

Annotation

Sub-cellular:
Anatomic:
Processes and Diseases: **Alveolar**, **Diffusion**
Medical:

Annotation

Sub-cellular:
Anatomic:
Processes and Diseases: **Circulatory Process**
Medical: **MESH:D010477 Perfusion**

Annotation

Sub-cellular:
Anatomic:
Processes and Diseases: **Muscle**, **Diffusion**
Medical:

Annotation

Sub-cellular:
Anatomic:
Processes and Diseases:
Medical: **MESH:D008928 Mitochondria**, **MESH:D008660 Metabolism**

other information

Gas exchange and transport

time-step size:

Model entities (Model Parameter)

Figure 29 Gas exchange and transport model annotations

1.4 PUBLIC KNOWLEDGE

This section summarizes COPD-related information from public sources. The COPD-Knowledge base is searched with certain predefined queries to give access to this information (Figure 30).

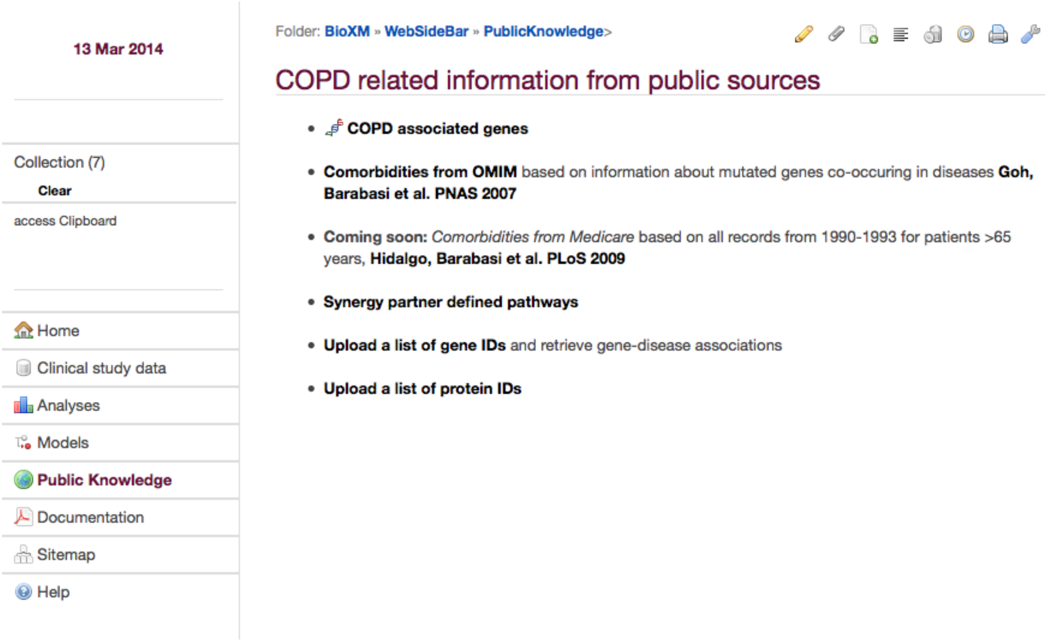

Figure 30 Public knowledge overview

Following the link "**COPD associated genes**" reveals a list of genes associated with COPD in public databases or by literature mining. Information related to these genes in the COPD-Knowledge base can be found by selecting various views in the left panel (Figure 31). Please note that the view "Gene – full functional information (slow)" might take up to several minutes to load.

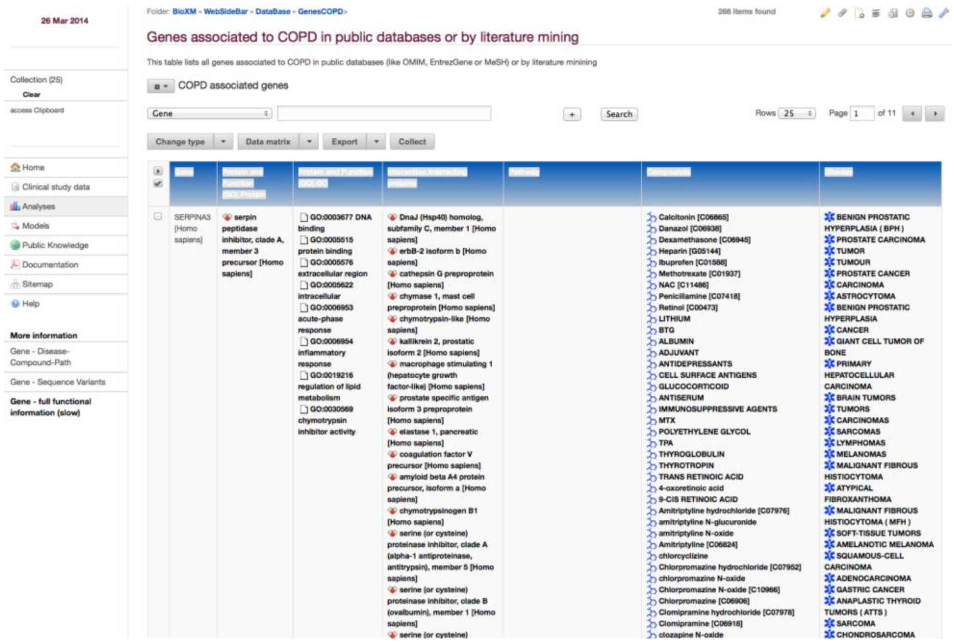

Figure 31 COPD related genes

Comorbidities from OMIM leads to disease – disease associations based on overlaps in gene – disease associations available from the OMIM database. I.e. two diseases are associated if they are known to be associated with at least one identical gene (Figure 32).

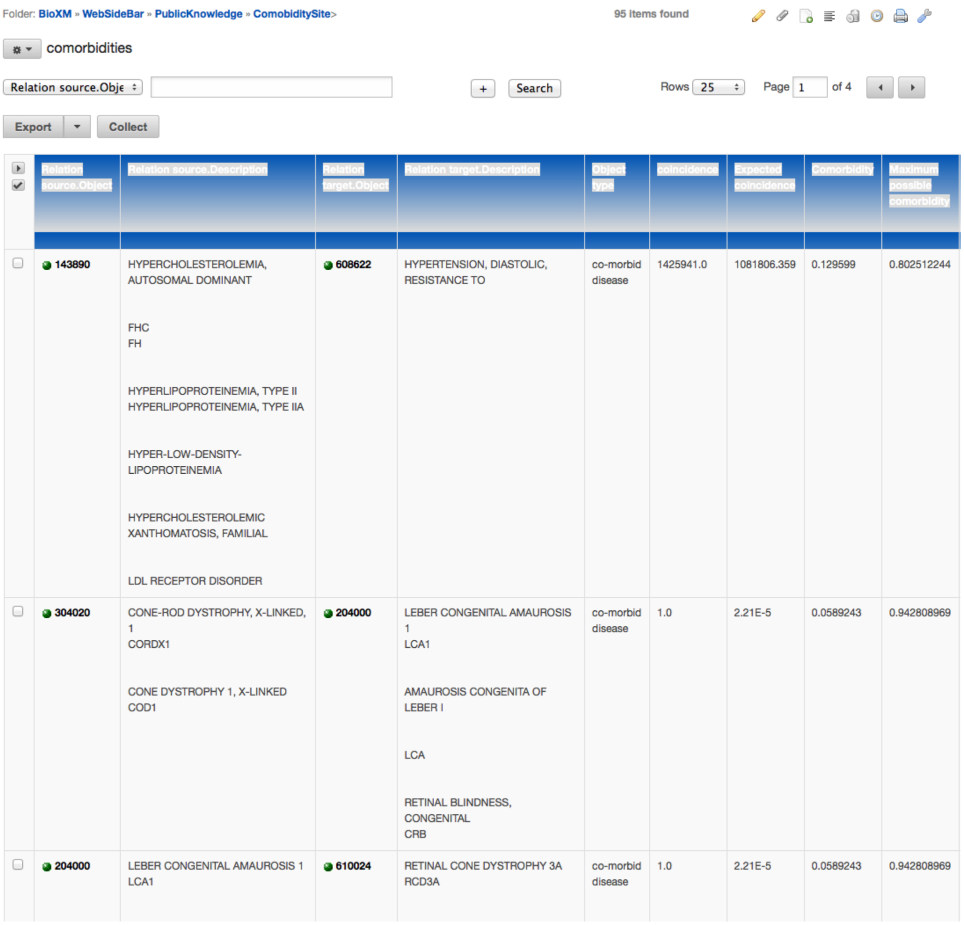

Relation source.Object	Relation source.Description	Relation target.Object	Relation target.Description	Object type	coincidence	Expected coincidence	Comorbidity	Maximum possible comorbidity
143890	HYPERCHOLESTEROLEMIA, AUTOSOMAL DOMINANT<br><br>FHC<br>FH<br><br>HYPERLIPOPROTEINEMIA, TYPE II<br>HYPERLIPOPROTEINEMIA, TYPE IIA<br><br>HYPER-LOW-DENSITY-LIPOPROTEINEMIA<br><br>HYPERCHOLESTEROLEMIC XANTHOMATOSIS, FAMILIAL<br><br>LDL RECEPTOR DISORDER	608622	HYPERTENSION, DIASTOLIC, RESISTANCE TO	co-morbid disease	1425941.0	1081806.359	0.129599	0.802512244
304020	CONE-ROD DYSTROPHY, X-LINKED, 1<br>CORDX1<br><br>CONE DYSTROPHY 1, X-LINKED<br>COD1	204000	LEBER CONGENITAL AMAUROSIS 1<br>LCA1<br><br>AMAUROSIS CONGENITA OF LEBER I<br><br>LCA<br><br>RETINAL BLINDNESS, CONGENITAL<br>CRB	co-morbid disease	1.0	2.21E-5	0.0589243	0.942808969
204000	LEBER CONGENITAL AMAUROSIS 1<br>LCA1	610024	RETINAL CONE DYSTROPHY 3A<br>RCD3A	co-morbid disease	1.0	2.21E-5	0.0589243	0.942808969

Figure 32 Comorbid diseases

Synergy partner defined pathways provides access to a list of manually curated signalling pathways involved in COPD and its systemic effects (Figure 33).

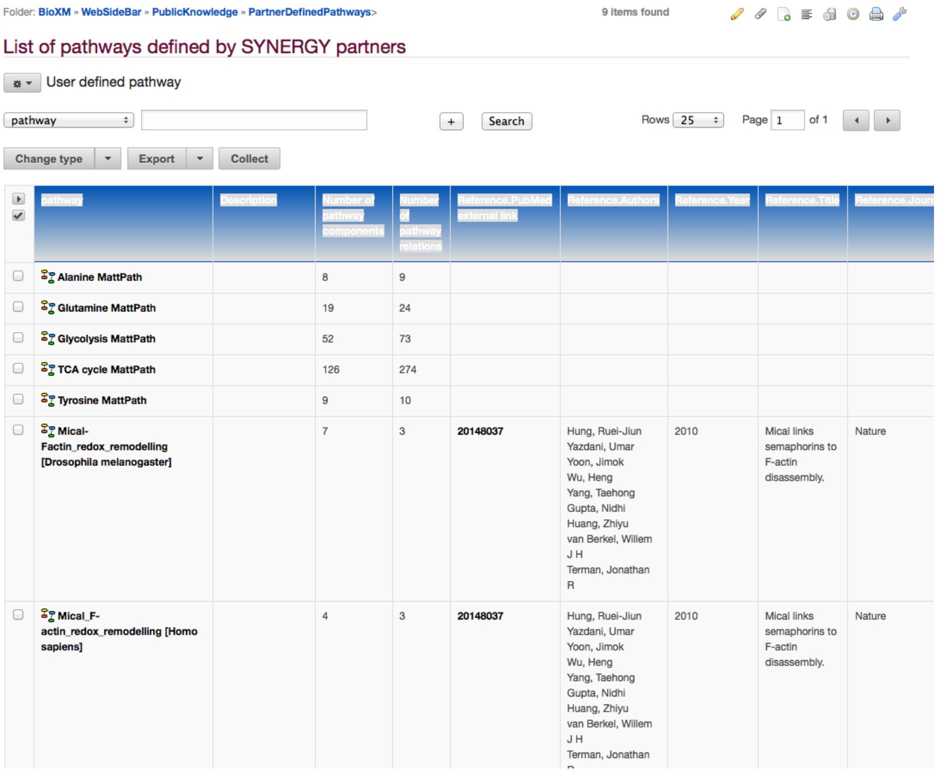

Folder: BioXM » WebSideBar » PublicKnowledge » PartnerDefinedPathways>   9 items found

List of pathways defined by SYNERGY partners

User defined pathway

pathway   + Search   Rows 25   Page 1 of 1

Change type   Export   Collect

pathway	Description	Number of pathway components	Number of pathway relations	Reference.PubMed external link	Reference.Authors	Reference.Year	Reference.Title	Reference.Jour
Alanine MattPath		8	9					
Glutamine MattPath		19	24					
Glycolysis MattPath		52	73					
TCA cycle MattPath		126	274					
Tyrosine MattPath		9	10					
Mical-Factin_redox_remodelling [Drosophila melanogaster]		7	3	20148037	Hung, Ruei-Jiun<br>Yazdani, Umar<br>Yoon, Jimok<br>Wu, Heng<br>Yang, Taehong<br>Gupta, Nidhi<br>Huang, Zhiyu<br>van Berkel, Willem J H<br>Terman, Jonathan R	2010	Mical links semaphorins to F-actin disassembly.	Nature
Mical_F-actin_redox_remodelling [Homo sapiens]		4	3	20148037	Hung, Ruei-Jiun<br>Yazdani, Umar<br>Yoon, Jimok<br>Wu, Heng<br>Yang, Taehong<br>Gupta, Nidhi<br>Huang, Zhiyu<br>van Berkel, Willem J H<br>Terman, Jonathan [illegible]	2010	Mical links semaphorins to F-actin disassembly.	Nature

Figure 33 COPD pathways

The “Public Knowledge” section also provides the possibility to **upload lists of gene or protein IDs** to retrieve the respective genes or proteins from the COPD-Knowledge base (Figure 34).

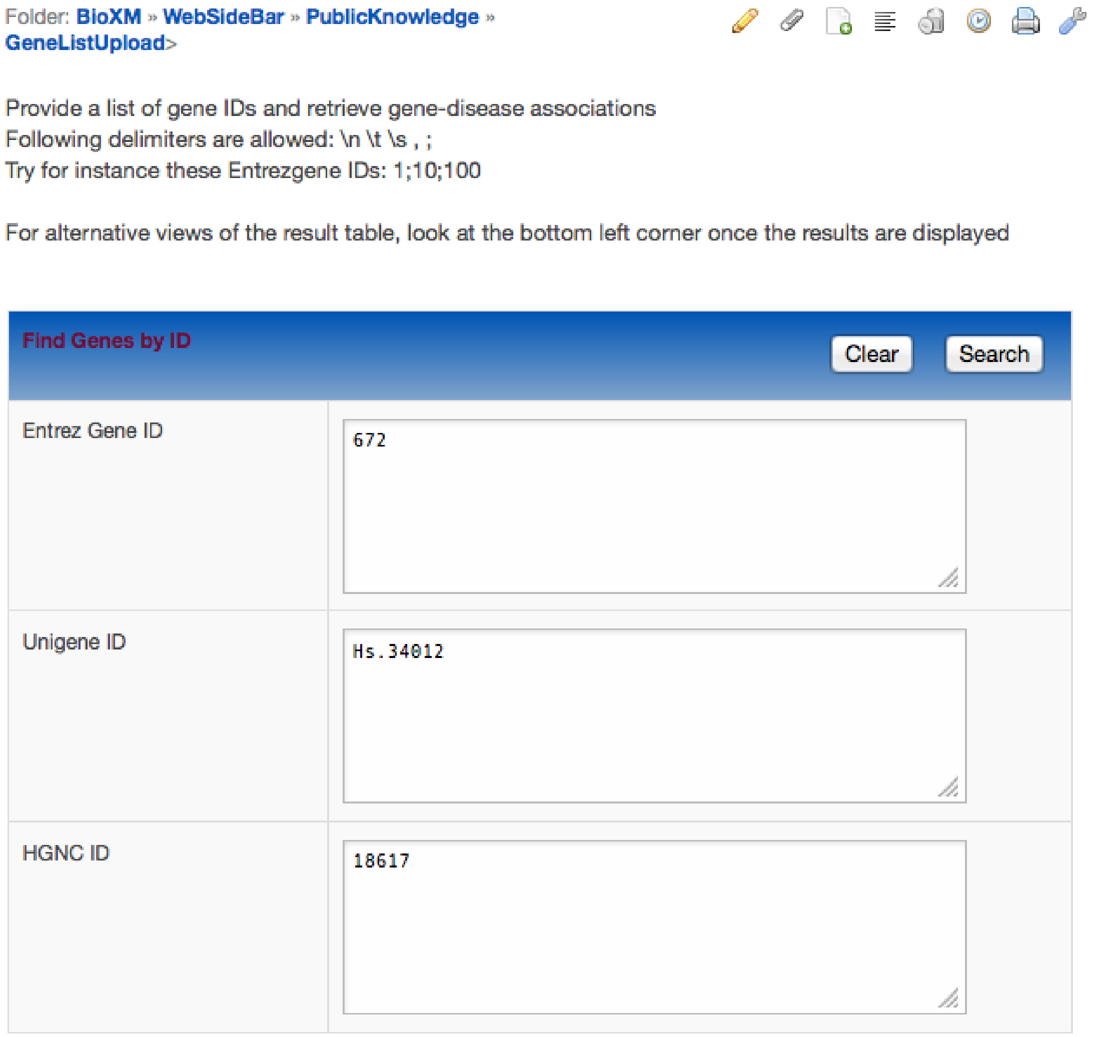

Figure 34 Upload of gene IDs

For genes, the disease associations are displayed in the result table (Figure 35).

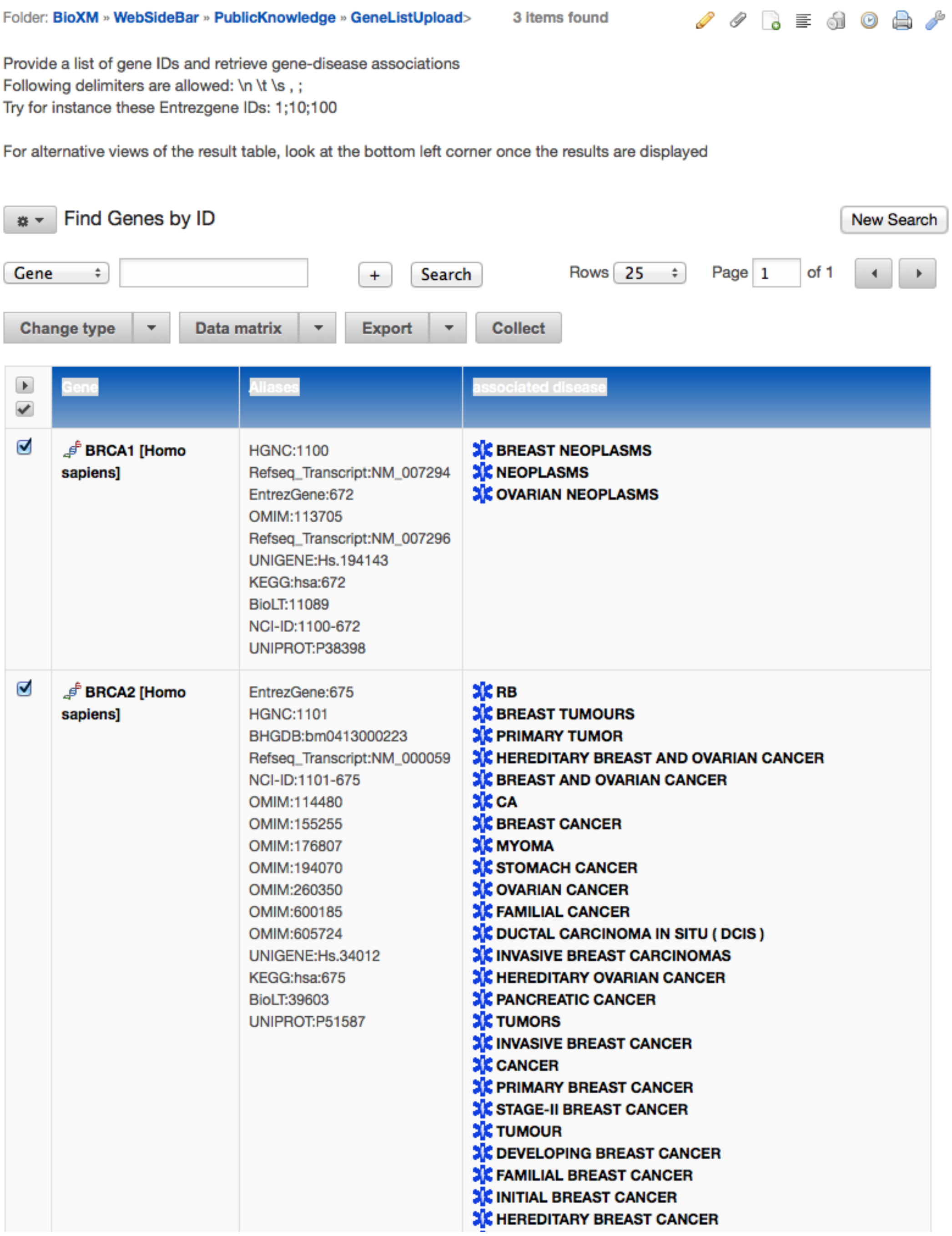

Figure 35 Gene result table

Further information related to these genes or proteins can then be found by selecting interesting genes/proteins with check marks and selecting appropriate menu points form the drop-down menues “Data matrix” or “Change type”. Gene/protein related information from public sources (like PPI, pathways) as well as information from the Biobridge and PAC-COPD studies (like Gene expression) can be retrieved (Figure 36 and Figure 37).

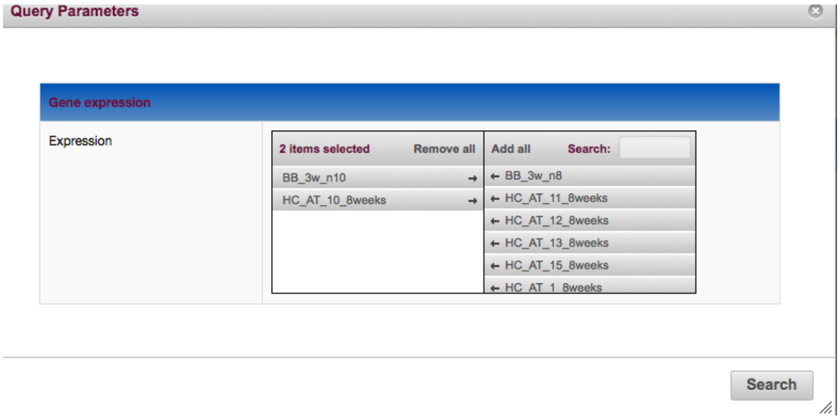

Figure 36 Selection of expression studies

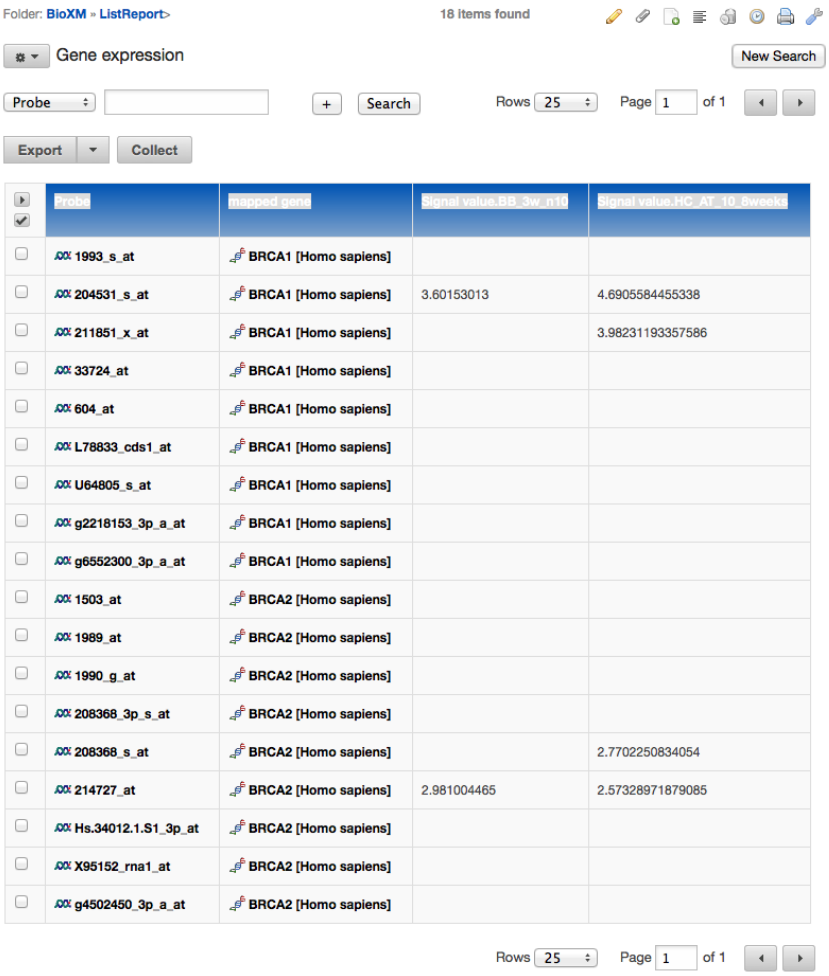

Probe	mapped gene	Signal value.BB_3w_n10	Signal value.HC_AT_10_8weeks
1993_s_at	BRCA1 [Homo sapiens]		
204531_s_at	BRCA1 [Homo sapiens]	3.60153013	4.6905584455338
211851_x_at	BRCA1 [Homo sapiens]		3.98231193357586
33724_at	BRCA1 [Homo sapiens]		
604_at	BRCA1 [Homo sapiens]		
L78833_cds1_at	BRCA1 [Homo sapiens]		
U64805_s_at	BRCA1 [Homo sapiens]		
g2218153_3p_a_at	BRCA1 [Homo sapiens]		
g6552300_3p_a_at	BRCA1 [Homo sapiens]		
1503_at	BRCA2 [Homo sapiens]		
1989_at	BRCA2 [Homo sapiens]		
1990_g_at	BRCA2 [Homo sapiens]		
208368_3p_s_at	BRCA2 [Homo sapiens]		
208368_s_at	BRCA2 [Homo sapiens]		2.7702250834054
214727_at	BRCA2 [Homo sapiens]	2.981004465	2.57328971879085
Hs.34012.1.S1_3p_at	BRCA2 [Homo sapiens]		
X95152_rna1_at	BRCA2 [Homo sapiens]		
g4502450_3p_a_at	BRCA2 [Homo sapiens]		

Figure 37 Gene expression experiments for selected genes

1.5 DOCUMENTATION

This section (Figure 38) provides the detailed documentation for the integrated data. Currently only data from the BioBridge study have been integrated.

In the **Synergy-COPD website** you can find detailed information about the project and related documentation.

Here you can download the deliverables of the BioBridge project providing a detailed description of data produced in the BioBridge study:

- **D3 - Identification of Relevant Datasets from the Public Domain**
- **D6 -Implementation of parameter estimation tools and 1rst prototype of optimal design tools**
- **D8 -Data integration**
- **D9 -SBML import-export to MathModelica Systems Biology**
- **D10 - Providing an additional set of molecular components based on statistical modelling**
- **D11 - First prototype of MathModelica Systems Biology**
- **D14 - Experimental Validation**
- **D15 - First prototype of the BioBridge portal**
- **D17 - Data integration report: interfaces and formats**

Figure 38 Downloadable documentation for the BioBridge project
